# Impact of Sex, Gonadectomy, and Repeated Restraint Stress on Gut Microbiome in Mice

**DOI:** 10.1007/s12035-025-05305-6

**Published:** 2025-11-19

**Authors:** Chahrazed Mekadim, Jakub Mrázek, Martin Vodička, Peter Ergang, Kateřina Olša Fliegerová, Tiziana Maria Mahayri, Kallayanee Chawengsaksophak, Jiří Pácha

**Affiliations:** 1 Laboratory of Anaerobic Microbiology, Institute of Animal Physiology and Genetics, Czech Academy of Sciences, V.V.I., Prague, Czech Republic; 2 Laboratory of Epithelial Physiology, Institute of Physiology, Czech Academy of Sciences, V.V.I., Prague, Czech Republic; 3 Department of Physiology, Faculty of Science, Charles University, Prague, Czech Republic; 4 Laboratory of Cell Differentiation, Institute of Molecular Genetics, Czech Academy of Sciences, V.V.I., Prague, Czech Republic

**Keywords:** Gut–brain axis, Gut microbiota, Stress, Sex difference, Gonadectomy, Neurogastroenterology

## Abstract

Recently, the bidirectional connection between the gastrointestinal microbiota and the brain has gained interest in many research studies. Findings have highlighted the potential role of stress and sex hormones in modulating the gut microbiome. To our knowledge, no study has investigated the effect of sex hormone perturbations on the gut microbiota in response to stress. To understand how stress may alter the gut microbiota differently depending on sex, gonadectomized and sham-operated male and female mice were subjected to 2 h of daily restraint stress for seven consecutive days. Body weight and plasma level of corticosterone were evaluated. Bacterial diversity and composition of colon and cecum were analyzed by sequencing of 16S rRNA gene. The bacterial communities were strongly altered by stress in the colon than in the cecum. A profound dysregulation of several metabolic and functional pathways was observed in sham mice. Alterations in the gut microbiome diversity and its functional pathways due to stress were more pronounced in males than in females. The present results provide potential sex-specific biomarkers and novel metabolic signatures in the gut microbiota related to stress disorders which may be used as potential targets in diagnostic and therapeutic approaches in neurogastroenterological diseases.

**Graphical Abstract:**

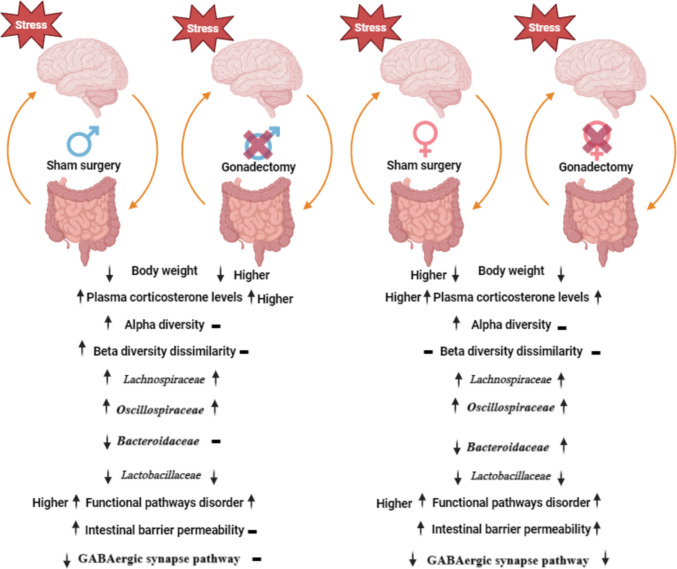

**Supplementary Information:**

The online version contains supplementary material available at 10.1007/s12035-025-05305-6.

## Introduction

The gut microbiota is a community of microorganisms, including bacteria, fungi, protozoa, archaea, and viruses that reside in the host gut [1–3]. Host-related factors, such as body weight, age, sex, and genetics, may shape the gut microbiome composition [4–7]. In particular, several studies have shown sex-dependent differences in the gut microbiota composition in both animals and humans [8–13], and sex hormones have been hypothesized to be responsible for these differences [10, 14, 15].

Numerous studies have shown that gut microbial communities play an essential role in host health and well-being, including brain development and function, neural metabolism, and mental health through the gut–brain axis [16–21]. The microbiota–gut–brain axis is a bidirectional process using several different mechanisms of communication [22]. Microorganisms produce many compounds with neurotransmitter or hormonal activity that affect distal sites of the host body such as the neuroendocrine system and the brain and regulate many physiological processes and behavioral responses [23–25]. Moreover, many studies have reported the association between gut microbiota dysbiosis with neurological diseases and mental disorders [26–30]. The transmission of stress-induced behavioral alterations [31] and some physiological changes in the gastrointestinal tract, including increased expression of proinflammatory cytokines and increased gut serotonin content [32, 33] from stressed to non-stressed recipients, was observed in rodent models after fecal microbiota transplantation (FMT). On the other hand, no physiological effect of stress was observed in germ-free mice [34] and in mice treated with an antibiotic cocktail [32]. In addition, one study revealed that FMT of healthy donors can improve stress-induced behavioral changes and changes in neuroactive substances in Wistar rats, but only if the vagus nerve is not damaged, which approves the influence of the gut–brain axis response [35]. This is evidence of the association of the gut microbiota with stress-induced disorders in host physiology. However, it is unclear how gut microbiota is involved in the development of these disorders. The composition of microbial communities varies along the different segments of the gastrointestinal tract (ileum, cecum, and colon) [36, 37]. Furthermore, innervations of the enteric nervous system with the gastrointestinal tract differ across its different regions [38]. Notably, Shaler et al. showed that gut microbiome-response to stress exhibited distinguished changes at different intestinal compartments [30]. Chronic and acute stress results in pronounced alterations in the gut microbial community and function [39–41] and is a trigger or risk factor for numerous diseases ranging from gastrointestinal inflammation and metabolic disorders to cardiovascular, dermatological, and psychiatric diseases [42]. These findings suggest that the impact of stress on the disorders might be, at least partially, due to changes in the gut microbiota [43]. However, most studies have been conducted using male animals, although sex-specific differences in stress response can be found at all stages of life [44–50]. Recent studies have also revealed sex-based differences in the stress-induced perturbation of gut microbial composition [51–60]. This sexual dichotomy contributes to differences in behavior between males and females via a sex-dependent microbiota–gut–brain axis [57, 61]. Male rodents exposed to stress showed higher emotional vulnerability, alterations of gut microbiota, and inflammatory system than females [52–54], and perturbations of sex hormones are associated with changes in the gut microbiota [10, 62–64]. To our knowledge, no study has investigated the effect of sex hormone perturbations on the gut microbiota in response to stress. Therefore, the aim of this study was to assess the interaction of sex and repeated stress on the gut microbiota in control and gonadectomized (GNX) mice. Our proposed hypothesis is the potential effect of sex hormone perturbation on the response of the gut microbiome to stress in a sex-dependent manner. First, we evaluated the effect of stress on body weight, plasma corticosterone level, and the gut microbiota of male and female mice, then, we demonstrated sex differences in gut microbiota composition and diversity in gonadectomized and sham-stressed and unstressed animals, and finally, we identified functional changes of various metabolic pathways associated with altered gut microbiota due to stress.

## Materials and Methods

### Ethics Approval Statement

All procedures were performed in concordance with the European legislation Directive 2010/63/EU on protecting animals for scientific purposes and the Czech legislation Act on the Protection of Animals from Cruelty No. 246/1992. Animal experiments were approved by the Committee for the protection and use of experimental animals of the Institute of Physiology, Czech Academy of Sciences (40–2022-P).

**Fig. 1:**
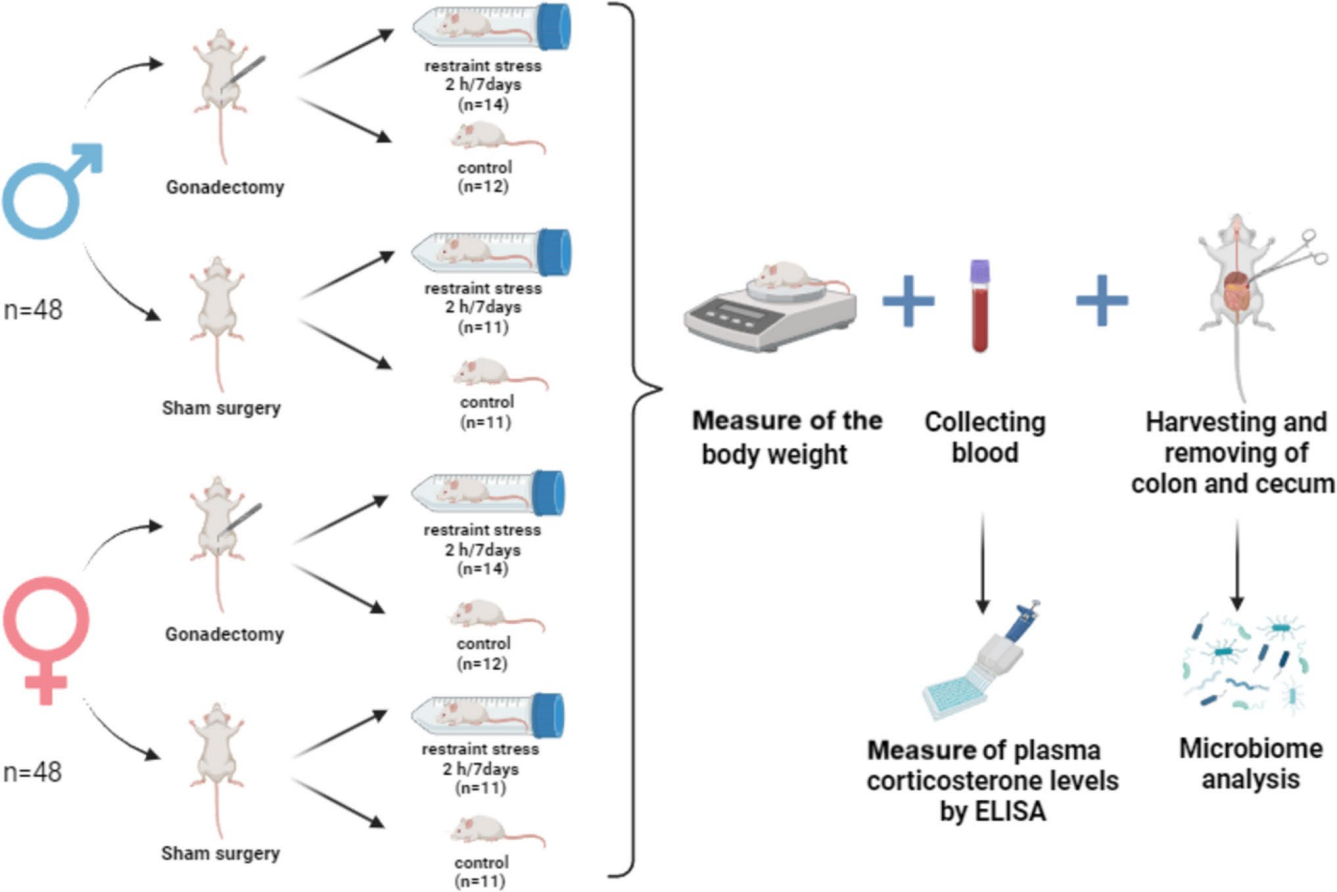
Experimental procedures. Experimental groups and number of animals in the respective experimental groups. Gonadectomy (GNX) or sham surgery (sham) was performed on 5-week old male and female mice. At 8 weeks of age, the mice were subjected to repeated restraint stress for 2 h daily for seven consecutive days (stress) or left undisturbed in their home cages (control). The restraint stress was performed between 9:30 a.m. and 3:30 p.m. After the last stress session, mice were deeply anesthetized in isoflurane vapors (4–5%) and weighted; and blood was collected and centrifuged; and plasma was used for measure of plasma corticosterone levels by ELISA. Colon and cecum were removed and used for microbiome analysis

Female and male BALB/c mice (*n* = 48 for each sex) were purchased from Charles River (Germany) for delivery at 4 weeks of age and were given 1 week to acclimate to the animal facility prior to surgeries. At the age of 5 weeks, the bilateral gonadectomy (GNX) or sham surgery was performed in both sexes. Ovariectomy was performed dorsally using a single transverse incision at the level of the third thoracic vertebra. The subcutaneous tissues and muscles were carefully separated, and a small incision was made on each side of the peritoneum to identify and remove the ovaries. Orchiectomy was performed via a ventral approach. A single transverse incision was made in the lower abdominal skin, followed by a second incision along the linea alba, allowing for the removal of the testes. In sham surgeries, the ovaries or testes were exposed but left intact. The presence or absence of gonads was visually verified after the animals were sacrificed. Following the surgery, all GNX- and sham-operated mice of the same sex were housed together in groups (4–5 per cage) and were allowed 3 weeks for recovery. At the age of 8 weeks, half of the GNX mice and half of the sham mice were pseudorandomly assigned to a stress protocol consisting of 2 h of restraint stress per day for seven consecutive days. The restraint stress was applied by placing the animal into falcon tube equipped with ventilation holes as in our previous study [65]. The restraint stress was applied in the light phase of day (randomly between 9:30 a.m. and 3:30 p.m.); all experimental groups underwent the stress procedure at comparable times to minimize potential circadian influences. To minimize stress in the control group, stressed mice were housed separately from control animals (2–3 per cage), and the stress protocol took place in a different room (the mice were transferred back to the animal room after each stress session). Control mice remained undisturbed in their home cages (Fig. 1). This procedure ensured that at least one animal from each original cage was distributed across all four experimental groups, minimizing potential “cage effect.” The mice were housed on a 12:12-h light:dark cycle with food (Altromin 1414) and water available ad libitum and lights off at 6 p.m.

After the last stress session, mice were deeply anesthetized in isoflurane vapors (4–5%) and weighed, and blood was collected by cardiac puncture into K_3_EDTA-coated tubes (Sarstedt, Nümbrecht, Germany) and centrifuged at 2000 g for 10 min at 4 °C, and plasma was stored at − 80 °C prior to testing. Immediately after the blood collection, the mice were decapitated. Approximately 0.5-cm long pieces of distal colon and cecum, including the contents, were removed, frozen in liquid nitrogen, and stored at − 80 °C for further analyses. All mice were sacrificed between 12 p.m. and 4 p.m. The level of plasma corticosterone was measured using Corticosterone rat/mouse ELISA KIT (AR E-8100, LDN GmbH, Nordhorn, Germany) according to the manufacturer’s instructions. Body weight and plasma level of corticosterone were analyzed using GraphPad Prism 6 software (GraphPad, La Jolla, CA, USA) and presented as the means ± SEM values. One-way or two-way analysis of variance (ANOVA) followed by Sidak’s post hoc test was performed to examine statistical significance in multiple group data sets. The *P* values less than 0.05 were considered to indicate statistical significance.

#### DNA Extraction and 16S rRNA Gene Sequencing

Total genomic DNA was extracted from the colon and cecum of each animal using the QIAamp® PowerFecal® Pro DNA Kit (Qiagen, Germany) according to the manufacturer’s instructions. The eluted DNA was stored at − 20 °C until further use for next-generation sequencing. Briefly, the extracted DNA was used as a template for preparation of amplicons from the V4V5 region of the 16S rRNA gene using the primer pair: BactB-F (GGATTAGATACCCTGGTAGT) and BactB-R (CACGACACGAGCTGACG). Libraries were prepared from the purified amplicons using NEBNext Fast DNA Library Prep Set kit (New England Biolabs, USA) according to Milani et al. [66]. The sequencing was then performed on the Ion Torrent platform (Thermo Fisher Scientific, USA) as described previously by Mekadim et al. [67].

#### Microbiome Analysis and Statistical Analysis

A total of 8,360,456 sequences were obtained: 4,914,555 from cecum samples and 3,445,901 from colon samples of all mice. The obtained sequences in FASTQ format were analyzed using the QIIME 2 version 2022.2 pipeline [68]. Briefly, quality filtering, chimera checking, and trimming of sequences were performed by the DADA2 [69]. Then, the obtained amplicon sequence variants (ASVs) were taxonomically classified using VSEARCH [70] based on SILVA database (version 138) with a 99% threshold of similarity. The rarefaction was performed based on the sequence depth (depth in cecum = 5956 and in colon = 9735) to normalize the data (Fig. S22). We ensured to choose the lowest sequence depth to include all sequenced samples and still a large frequency per sample. The selected sequenced depths for cecal and colonic microbiome are adequate and capture the most microbial diversity of all samples.

The alpha diversity was determined using Shannon, Chao1, and Pielou’s Evenness indices based on the Kruskal–Wallis test. Principal coordinate analysis (PCoA) was obtained based on Bray–Curtis distance (beta diversity). The box plots for the indices of diversity and the 2-dimensional PCoA plots were generated in R-Studio (http://www.rstudio.com/) using ggplot2 packages [71]. Ellipses mark 95% of confidence for each group and *P* ≤ 0.05 was considered statistically significant. Bray–Curtis dissimilarity distance was calculated, and the significances between groups were evaluated using permutational multivariate analysis of variance (PERMANOVA) implemented in adonis2 function from vegan R package (2.6) [72]. The linear discriminant analysis with effect size (LEfSe) tool [73] was performed based on Kruskal–Wallis test and the pairwise Wilcoxon test to identify genera with significantly different relative abundances between groups with *α* value of 0.05 and threshold value at 2.0.

#### Bacterial Predictive Functionality

PICRUSt2 v2.5.2–0 [74] was applied for functional pathway prediction using the KEGG database. The STAMP program [75] was utilized for statistical analysis by using non-corrected type two-sided Welch’s *t* test, with a confidence interval (CI) of 0.95, and *P* < 0.05 was considered to be statistically significant. No correction for multiple comparisons was applied. Consequently, the likelihood of type I error is increased, potentially leading to statistically significant findings that may be false positives. Readers should consider this limitation when interpreting the results. The correlation analysis between differentially significant bacterial genera and functional pathways was carried out using the Spearman rank correlation test (Spearman’s rank correlation test, *P* < 0.05).

## Results

### Effect of Stress and Gonadectomy on Weight and Plasma Corticosterone (CORT) Levels

**Fig. 2:**
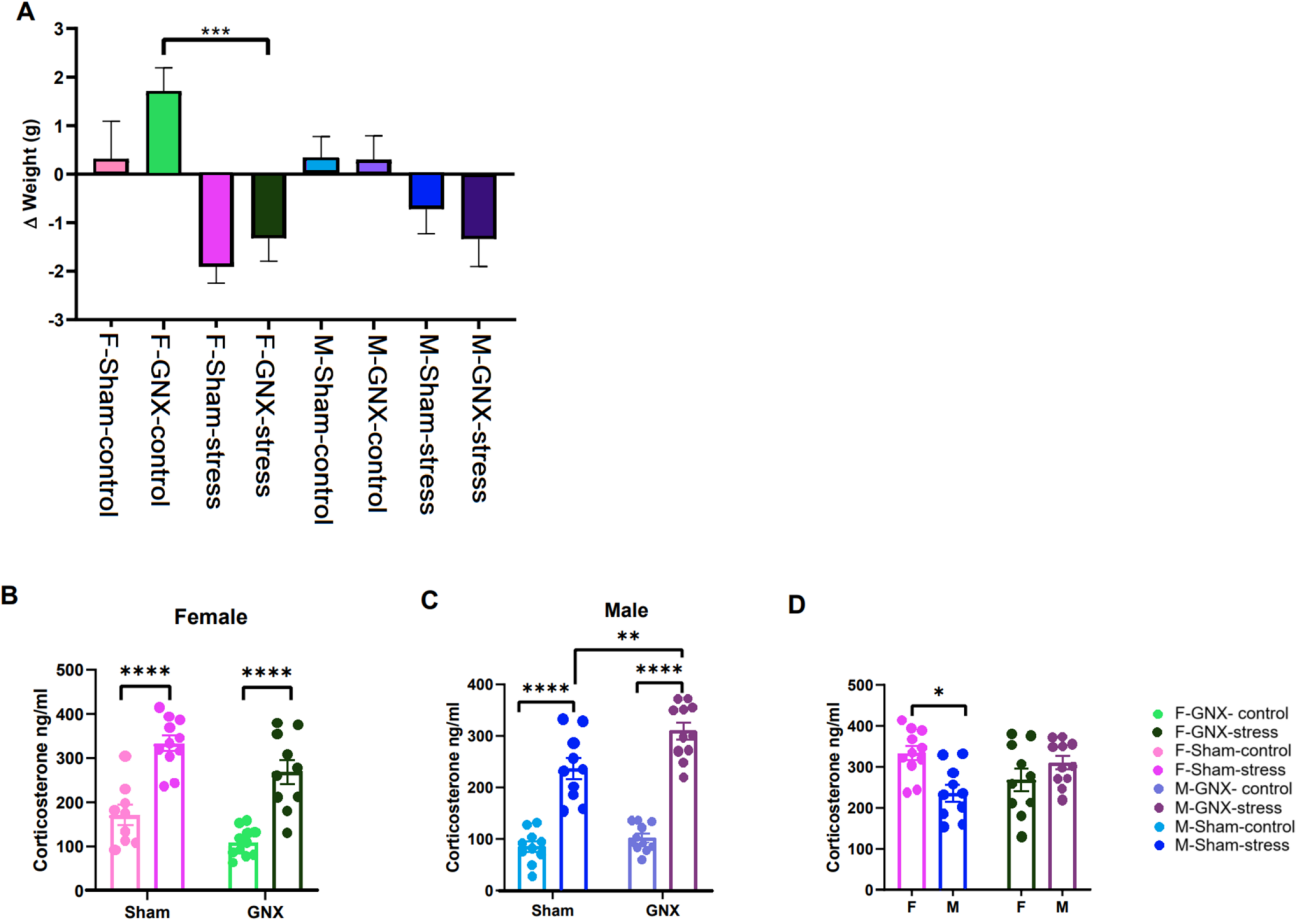
Effect of sex, gonadectomy, and stress on changes in body weight (**A**) and plasma levels of corticosterone (**B–D**). The data were analyzed using one-way (**A**) or two-way (**B–D**) ANOVA followed by post hoc test. Statistical significance: *P < 0.05; **P < 0.01; ***P ≤ 0.001; ****P ≤ 0.0001. F, female; M, male; GNX, gonadectomized; Sham, sham-operated; Stress, repeatedly restrained for 1 week, Control, unstressed animals

Statistical analysis using one-way ANOVA revealed significant differences of experimental conditions on weight changes of mice [*F*(7,80) = 5.436, *P* < 0.0001] between the start and end of the 7-day stress protocol. Furthermore, post hoc analysis of this main effect showed that the group of GNX females challenged with stress had a significantly different weight change relative to unstressed GNX females (Fig. 2A).

Plasma corticosterone (CORT) levels of mice exposed to the repeated restraint stress were increased (Fig. 2). In females, the two-way ANOVA revealed a significant effect of stress [*F*(1,37) = 66.81, *P* < 0.0001] and gonadectomy [*F*(1,37) = 10.25, *P* = 0.0028] but no significant interaction between these two factors. Post hoc analysis showed that repeated stress increased CORT levels in both sham and GNX groups compared to the respective unstressed controls (Fig. 2B). Similarly, the two-way ANOVA revealed in males the effect of stress [*F*(1,38) = 158.3, *P* < 0.0001] and gonadectomy [*F*(1,38) = 10.08, *P* = 0.0030], but no interaction between them. Post hoc analysis showed that stress upregulated CORT in both sham and GNX groups compared to unstressed controls. In addition, following the last stress session, CORT reached significantly higher plasma levels in gonadectomized males than in their sham-operated counterparts (Fig. 2C). Moreover, a significant interaction between sex and gonadectomy was observed for the CORT levels [*F*(1,38) = 11.57, *P* = 0.0016], and the post hoc analysis revealed a difference between males and females in sham-stressed mice but not in GNX-stressed mice (Fig. 2D).

### Effect of Stress, Sex, and Gonadectomy on Gut Microbiome Diversity

#### Alpha Diversity

**Fig. 3:**
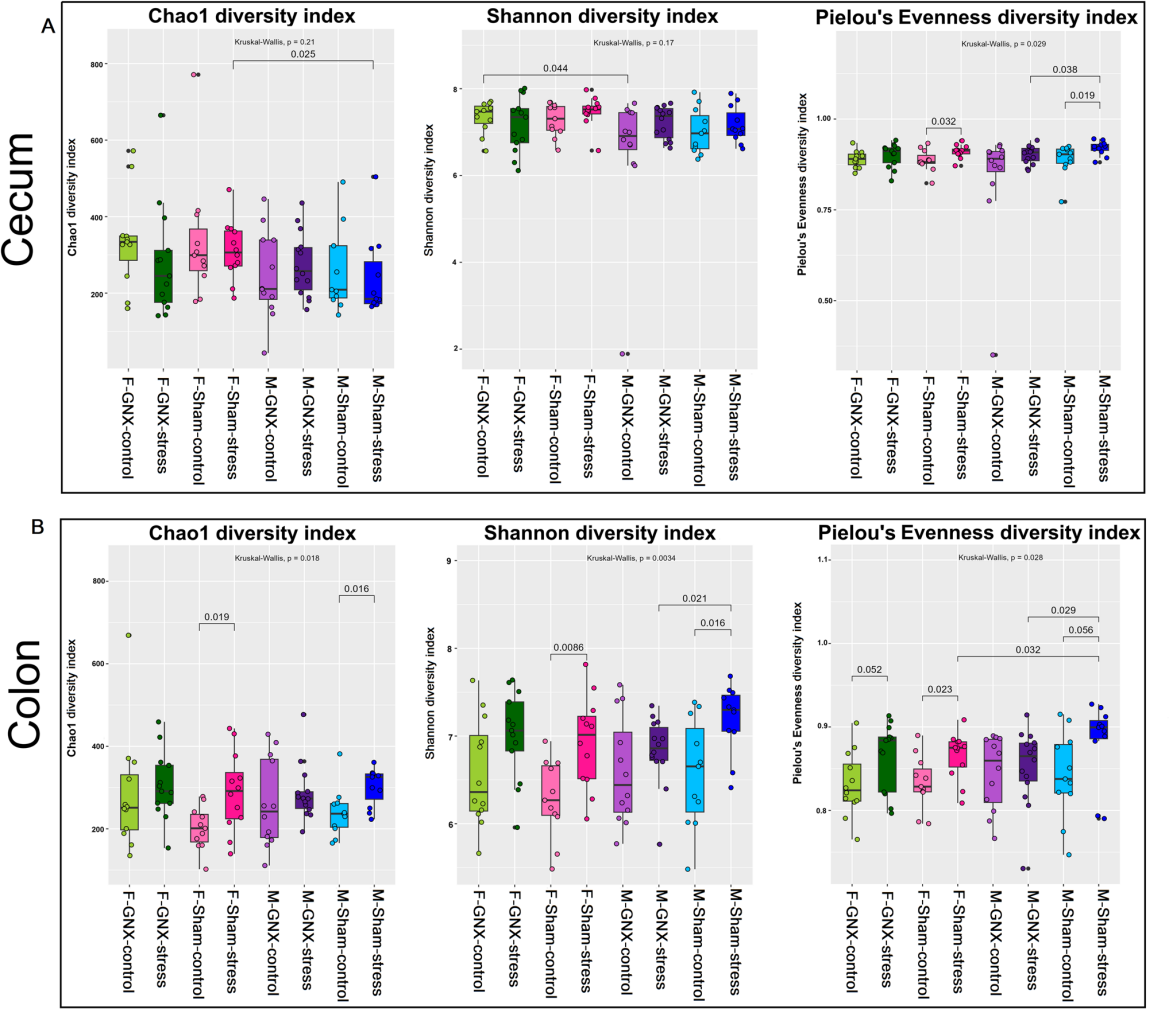
Effect of sex, gonadectomy, and stress on the alpha diversity. Boxplots illustrating Chao1, Shannon, and Pielou’s Evenness diversity indices in bacterial community of cecum (**A**) and colon (**B**) of different groups of mice. *P* ≤ 0.05 was considered statistically significant based on the Kruskal–Wallis test

The results of alpha diversity using the Chao1, Shannon, and Pielou’s evenness indices are presented in the boxplot graph (Fig. 3).

In the cecum (Fig. 3A), stress altered significantly bacterial evenness of sham stressed in both sexes (females *P* = 0.032, males *P* = 0.019). Stress did not have any significant effect on all diversity indices in gonadectomized mice of both sexes. No significant difference in the effect of stress on bacterial richness was observed between GNX and sham mice in both sexes (*P* > 0.05). However, stress impact on bacterial evenness was significantly higher in sham-stressed males than in GNX-stressed counterparts (*P* = 0.038). A sex-difference effect of stress on bacterial richness (Chao1) was only observed in sham-stressed mice (*P* = 0.025).

In the colon (Fig. 3B), stress resulted in differences in Chao1 and Shannon indices in sham males (*P* = 0.016) and in all diversity indices (Chao1, Shannon, and evenness) in sham females (with *P* values of 0.019, 0.0086, and 0.023 respectively). Gonadectomy prevented the effects of stress on all diversity indexes in all mice of both sexes, while the Shannon and evenness indices were significantly higher in sham-stressed males in comparison to GNX-stressed counterparts (*P* = 0.021 and *P* = 0.029, respectively). Noticeably, a significant sex-difference effect of stress on bacterial evenness was observed between males and females of sham-stressed animals (*P* = 0.032). This difference was only observed in the evenness but not in the bacterial richness.

#### Beta Diversity (PCoA)

**Fig. 4:**
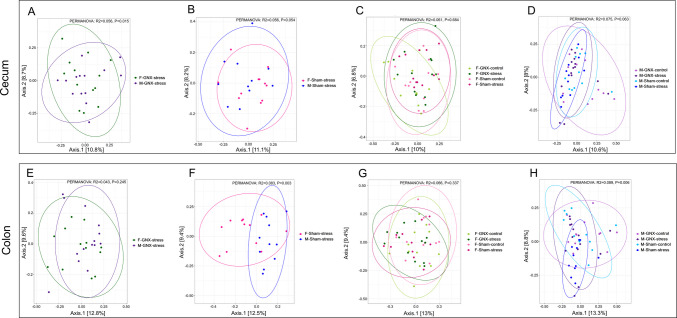
Effect of sex on the beta diversity of the gut microbiome of stressed mice. Principal coordinate analysis (PCoA) plots based on the Bray–Curtis distance showed distinct clusters in the microbiome of cecum (**A**, **B**, **C**, **D**) and colon (**E**, **F**, **G**, **H**) of gonadectomized stressed mice (**A**, **E**), sham stressed mice (**B**, **F**), all females (**C**, **G**), and all males (**D**, **H**). *P* ≤ 0.05 was considered statistically significant. List of all *P* values for pairwise PERMANOVA tests are presented in Table S1

Principal coordinate analysis (PCoA) based on Bray–Curtis distance was performed on colon and cecum microbiome separately to compare the diversity between different gender and treatment groups (Fig. 4, S1, S2, and Table S1).

Stress altered both the cecum (*P* = 0.043) and the colon (*P* = 0.004) microbiome composition in sham male, but not in female (*P* > 0.05) mice (Fig. S1 B, E, Fig. S2 B, E). Such effects of stress were abolished in gonadectomized male mice as shown by the lack of difference in cecum and colon microbiome composition between M-GNX-control and M-GNX-stress (*P* > 0.05) (Fig. S2 A, D). Sex-difference effects of stress were observed in the cecum of GNX-stressed mice (*P* = 0.015) (Fig. 4A) but not in the colon, and in the colon of sham-stressed mice (*P* = 0.003; (Fig. 4F) but not in the cecum. Moreover, stress influences only the colonic microbiome of male mice (*P* = 0.006) (Fig. 4H) and not cecal microbiome (Fig. 4D). This effect was not observed in females (Fig. 4C, G).

### Effect of Stress, Sex, and Gonadectomy on the Microbiota Composition

At the phylum level (Fig. S3 and Tables S2, S3), the colonic and cecal microbiome of all mice was dominated by *Bacillota* (formerly called *Firmicutes*), followed by *Bacteroidota* (formerly called *Bacteroidetes*) (Fig. S3). *Bacteroidota* was more prevalent in the colonic microbiota of control groups compared to the stressed groups, in which *Bacillota* was the most abundant phylum.

**Fig. 5:**
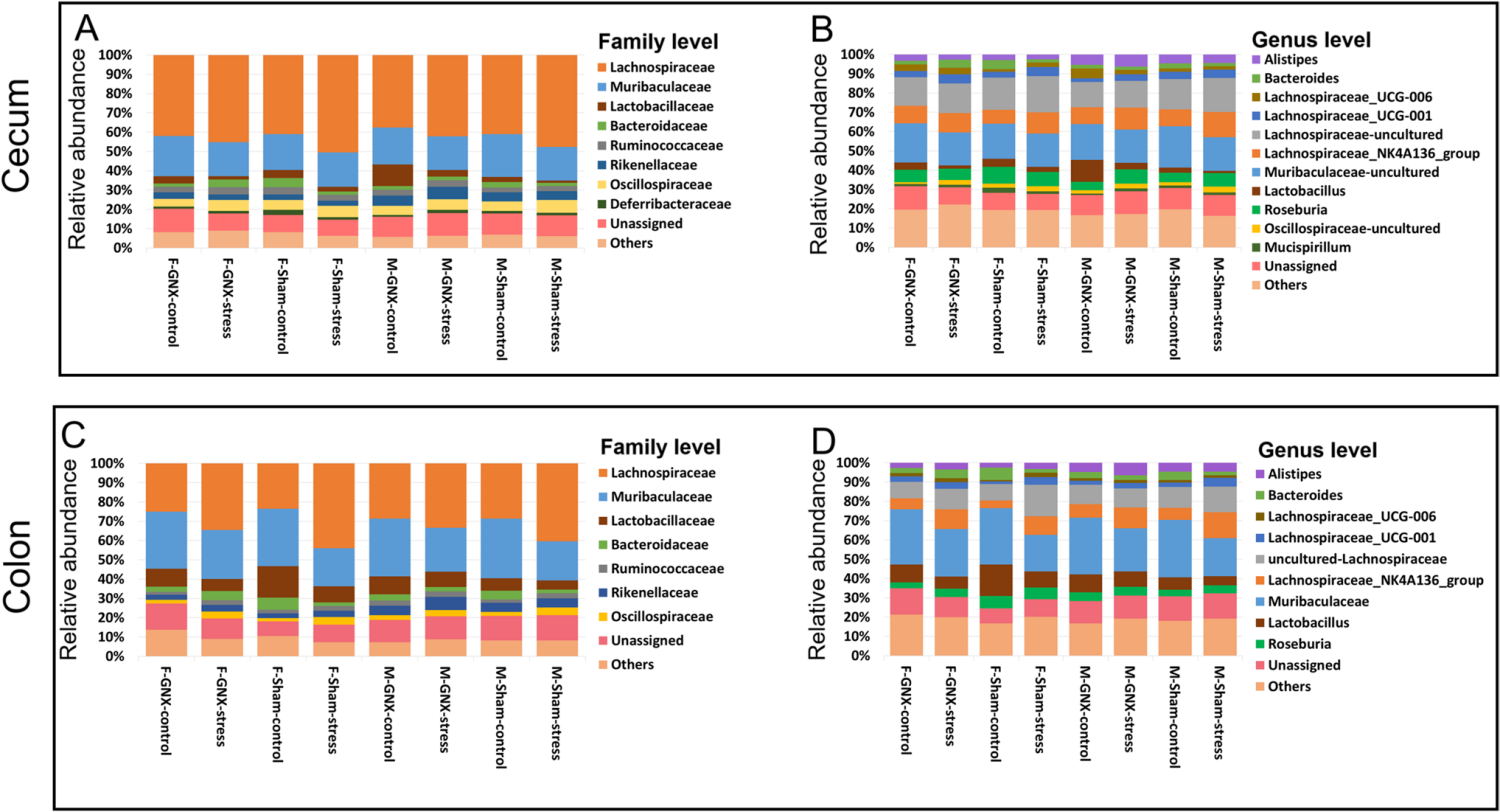
Effect of sex, gonadectomy, and stress on the gut microbiota composition. Relative abundance of bacterial populations at family (**A**, **C**) and genus (**B**, **D**) levels in microbiomes of cecum (**A**, **B**) and colon (**C**, **D**) of different groups of mice

At the family level (Fig. 5A, C**, **S21, and Tables S2, S3), *Lachnospiraceae* and *Oscillospiraceae* were more abundant, and *Muribaculaceae* and *Lactobacillaceae* less abundant in the colonic microbiota of all stressed mice compared to control mice. Typically, *Bacteroidaceae* were lower in the colonic microbiota of sham-stressed mice compared to control mice.

At the genus level (Fig. 5B, D and Tables S2, S3), *Lachnospiraceae*_NK4A136_group, uncultured *Lachnospiraceae*, and *Lachnospiraceae* UCG-001 were higher in the colonic microbiota of stressed mice compared to control mice. Unclassified *Muribaculaceae*, *Bacteroides*, and *Lactobacillus* were lower in the colonic microbiome of all stressed mice compared to control mice.

No major differences have been observed in the composition of the cecal microbiome of all mice, except for a lower abundance of *Lactobacillus* in stressed GNX male mice compared to the control GNX male mice.

The linear discriminant analysis with effect size (LefSe) was used to detect genera with significantly different relative abundances in each group in the cecal and colonic microbiome separately with alpha values of 0.05 and a threshold value of 2.0 on the logarithmic LDA scores for discriminative features.

### Bacterial Markers Related to Stress and Gonadectomy in the Gut Microbiota of Stressed Mice of Both Sexes

**Fig. 6:**
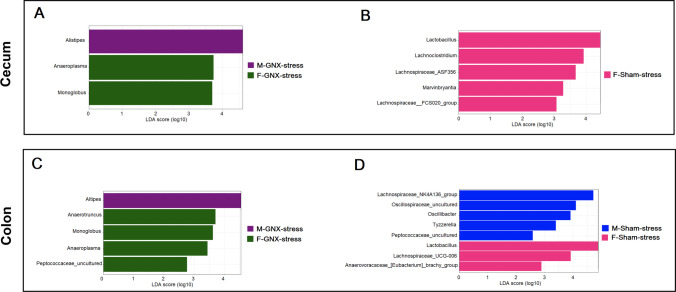
Sex-related bacterial markers in the gut microbiome of stressed mice. Linear discriminant analysis effect size (LEfSe) of taxa at genus level in the microbiome of cecum (**A**, **B**) and colon (**C**, **D**) between males and females of stressed gonadectomized mice (**A**, **C**) and between males and females of stressed sham mice (**B**, **D**) with alpha values of 0.05 and a threshold value of 2.0

In the microbiome of the colon and cecum (Fig. 6, S4, and S5), many genera were distinguished between different treatments and types of surgery in both sexes. In comparison based on stress treatment within the same surgery type and sex, more bacterial markers were detected in the colonic microbiome than in the cecal microbiome of sham-stressed mice (Fig. S4E, S5E). *Lachnospiraceae*_NK4A136_group and *Butyricicoccaceae*-UCG-009 were the common biomarkers in the colon of stressed sham mice in both sexes (Fig. S4E, S5E). Uncultured bacterium of *Rumiunococcaceae* family was the common biomarker in the colon of stressed GNX mice in both sexes (Fig. S4D, S5D). *Akkermansia* was the only biomarker in the cecal microbiome of sham-stressed males (Fig. S4 B). *Lachnospiraceae*_NK4A136_group was the common biomarker in the colonic and cecal microbiome of sham-stressed females (Fig. S5 B, E). Uncultured bacterium of *Ruminococcaceae* was the common bacterial signal in both the colon and cecum of male stressed GNX mice (Fig. S4 A, D). Uncultured bacterium of *Oscillospiraceae* was the common bacterial marker in both the colon and cecum of GNX-stressed females (Fig. S5 A, D). *Candidatus Arthromitus* and an uncultured bacterium of *Ruminococcaceae* were the shared biomarkers in colonic microbiome of both GNX- and sham-stressed males (Fig. S4 D, E). *Lachnospiraceae*_NK4A136_group and *Butyricicoccaceae*-UCG-009 were the common bacterial genera markers associated with the colonic microbiome of both GNX- and sham-stressed females (Fig. S5 D, E).

In comparison based on type of surgery within the stressed groups, higher number of biomarkers were identified in the colonic microbiome of sham-stressed male mice (Fig. S4 F). *Monoglobus* was the bacterial genus marker related to GNX-stressed females in both the colon and cecum (Fig. S5 C, F). *Lactobacillus* was the bacterial genus marker associated with GNX-stressed males in both colonic and cecal microbiome (Fig. S4 C, F). *Peptococcus* and uncultured bacterium of *Lachnospiraceae* were common bacterial markers correlated with sham-stressed males in both the colon and cecum (Fig. S4 C, F).

In comparison based on sex within the stressed groups, higher differences were observed in the colonic microbiome of sham-stressed mice (Fig. 6). *Alistipes* was the only biomarker for GNX-stressed males in both the colon and cecum (Fig. 6A, C). *Anaeroplasma* and *Monoglobus* were common biomarkers for GNX-stressed females in both colonic and cecal microbiome (Fig. 6A, C). *Lactobacillus* was the common biomarker for sham-stressed females in microbiome of both intestinal segments (Fig. 6B, D). *Lachnospiraceae*_NK4A136_group, uncultured bacterium of *Oscillospiraceae*,* Oscillibacter*, *Tyzzerella*, and uncultured bacterium of *Peptococcaceae* were bacterial genera markers in the colonic microbiome of sham-stressed males (Fig. 6D). However, no biomarker was detected in the cecal microbiome of the same group of mice (Fig. 6B).

### Effect of Stress, Sex, and Gonadectomy on the Functional Potential of the Gut Bacterial Community

PICRUSt2 (Phylogenetic Investigation of Communities by Reconstruction of Unobserved States) is a software tool used only for predicting functional abundances based on 16S rRNA gene sequencing data. In the present study, PICRUSt2 was used to determine sex differences in predicted functional pathways in the intestinal microbiota of mice in response to stress. Results showed large significant differences between the cecum and colon. Several distinguished functions indicated that the colonic microbiota is more affected by the stress than cecal microbiota in cecum.

The functional prediction analysis of the gut microbiota revealed the abundance of many microbial functional genes related to “metabolism,” “environmental information processing,” “genetic information processing,” “cellular processes and signaling,” “organismal systems,” and “human diseases” (Fig. S6–S20, Tables S4, S5).

The cecal microbiome of GNX-stressed female mice was significantly rich in genes correlated with “propanoate metabolism,” “mineral absorption,” “cationic antimicrobial peptide (CAMP) resistance,” “*Staphylococcus aureus* infection,” “ascorbate and aldarate metabolism,” “porphyrin metabolism,” “atrazine degradation,” “pinene, camphor, and geraniol degradation,” and “tyrosine metabolism,” while 16 significantly abundant functions were enriched in the cecal microbiome of control counterpart including “GABAergic synapse” (Fig. S6). “Chloroalkane and chloroalkene degradation,” “bacterial chemotaxis,” “glycerolipid metabolism,” and “two-component system” were significantly abundant functions in the cecal microbiome of sham-stressed females, while “GABAergic synapse” was also significantly enriched in the cecal microbiome of sham control females (Fig. S7). No significant results were revealed between GNX- and sham-stressed females in cecal microbiome. “Propanoate metabolism” and “bacterial chemotaxis” were the only two significantly abundant functions in the cecal microbiome of GNX-stressed males while “homologous recombination,” “taurine and hypotaurine metabolism,” and “lipoic acid metabolism” were significant functions identified in the cecal microbiome of control counterpart (Fig. S8). Thirty-six functions were significantly dysregulated in the cecal microbiome of sham males, with ten of them significantly increased after stress (Fig. S9). The regulation of 28 functions in the cecal microbiome was different between sham-stressed males and GNX-stressed males (Fig. S10). The results show a sex difference in the regulation of 38 pathways in the cecal microbiome of GNX-stressed mice (Fig. S11). “Pentose and glucoronate interconversions,” “cationic antimicrobial peptide (CAMP) resistance,” “ABC transporters,” “peroxisome,” “sulfur relay system,” “mineral absorption,” “glyoxylate and dicarboxylate metabolism,” and “porphyrin metabolism” were the only eight pathways significantly more frequent in the cecal microbiome of GNX females, while interesting pathways like “pathways in cancer,” “Huntington disease,” and “GABAergic synapse” were significantly expanded in the cecal microbiome of GNX males (Fig. S11). Similarly, the analysis shows the sex differences in the regulation of 23 pathways in the cecal microbiome of sham-stressed mice (Fig. S12). Pathways like “phosphotransferase system (PTS),” “glycolysis/gluconeogenesis,” and “fatty acid degradation” were significantly upregulated in cecal microbiome of sham females, while “Epithelial cell signaling in *Helicobacter pylori* infection,” “phenylalanine, tyrosine, and tryptophan biosynthesis,” and “fatty acid biosynthesis” were significantly increased in the cecal microbiome of sham males (Fig. S12).

The difference in functional regulation pathways was profound in the colonic microbiome. Thirty-eight different pathways were revealed in the colonic microbiome of GNX female mice. “Bacterial chemotaxis,” “flagellar assembly,” “two-component system,” “ABC transporters,” and “quorum sensing” were enhanced in the colonic microbiome of stressed GNX female mice (Fig. S13). Thirty-seven significant functional pathways were distinguished in the colonic microbiome of sham females, while six functional pathways of “flagellar assembly,” “styrene degradation,” “bacterial chemotaxis,” “lysine biosynthesis,” “biofilm formation,” and “secondary bile acid biosynthesis” were enhanced in the colonic microbiome of stressed sham females (Fig. S14). Genes related to “pyruvate metabolism” and “carbohydrate digestion and absorption,” “bacterial invasion of epithelial cells,” “serotonergic synapse,” and “nonribosomal peptides structures” were significantly higher in the colonic microbiome of stressed GNX females than in stressed sham females, in which “cell cycle-*Caulobacter*” was the only significant abundant pathway (Fig. S15). “Parkinson disease” and “chloroalkane and chloroalkene degradation” pathways were significantly more abundant in the colonic microbiome of GNX-stressed males compared to GNX control males (Fig. S16). Forty-nine variable significant pathways were detected in the colonic microbiome of sham males of which 13 belong to functional pathways related to “bacterial chemotaxis,” “flagellar assembly,” “two-component system,” “exopolysaccharide biosynthesis,” “cationic antimicrobial peptide (CAMP) resistance,” “secondary bile acid biosynthesis,” and others were significantly higher in the colon of stressed sham male mice (Fig. S17). In the comparison between the colonic microbiome of sham- and GNX-stressed male mice, 42 significant pathways were identified. The functional pathways of “bacterial chemotaxis,” “flagellar assembly,” “two-component system,” “exopolysaccharide biosynthesis,” “cationic antimicrobial peptide (CAMP) resistance,” “secondary bile acid biosynthesis,” and others were significantly increased in the colon of sham-stressed males while pathways of “ribosome,” “glycolysis/gluconeogenesis,” “GABAergic synapse,” and others were significantly enriched in the colon of GNX-stressed males (Fig. S18). The sex difference was evident in the colonic microbiome of GNX-stressed mice, where six variable pathways were differently regulated (Fig. S19). In the colon microbiome of sham-stressed mice, the sex difference was large in 44 functional pathways (Fig. S20).

### Correlations Between Differentially Abundant Genera and Altered Functional Pathways Induced by Stress and Gonadectomy

The Spearman correlation analysis for significantly different functions and microbes was performed to obtain the relationships between altered functional pathways and identified bacterial markers specific for each group in cecal and colonic microbiomes of males (Fig. S23) and females (Fig. S24). “Bacterial chemotaxis” and “propanoate metabolism” were strongly correlated with *Lachnospiraceae*_UCG-001 and an uncultured bacterium of *Ruminococcaceae* in the cecal microbiome of GNX-stressed males and negatively correlated with [Eubacterium]_coprostanoligenes_group in control counterpart (Fig. S23 A). *Akkermansia,* the only biomarker in the cecal microbiome of sham-stressed males (Fig. S4 B), was negatively correlated with most of predicted functional pathways (Fig. S23 B). “Parkinson’s disease” and “Fructose and mannose metabolism” were positively correlated with *Candidatus Arthromitus* and an uncultured bacterium of *Ruminococcaceae* in the colonic microbiome of GNX-stressed male mice and negatively correlated with uncultured bacterium of *Muribaculaceae* in the colonic microbiome of control counterpart (Fig. S23 D, Fig. S4 D). “C5-branched dibasic acid metabolism” and “thyroid hormone synthesis” were found to be positively correlated with *Lactobacillus*, which was differentially abundant in both cecal and colonic microbiomes of GNX-stressed males and negatively correlated with *Peptococcus* and an uncultured bacterium of *Lachnospiraceae*, common bacterial markers correlated with sham-stressed males in both the colon and cecum (Fig. S23 C, F, Fig. S4 C, F). In the colonic microbiome of sham-stressed males, “Huntington diseases” was positively correlated only with *Lachnospiraceae*_UCG-001, and “spinocerebellar ataxia” was positively correlated only with *Oscillibacter* (Fig. S23 E). “GABAergic synapse” was negatively correlated with uncultured bacterium of *Muribaculaceae*, *Lachnospiraceae*_NK4A136_group, *Muribaculum*, *Oscillibacter*, *Lachnospiraceae*_A2, and *Butyricicoccaceae*_UCG-009 and positively correlated with *Candidatus Arthromitus*, *Tyzzerella*, *Lactobacillus*, and *Peptococcus* (Fig. S23 E, F). *Lachnospiraceae*_NK4A136_group, uncultured bacterium of *Ruminococcaceae*, and *Butyricicoccaceae*_UCG-009 were positively correlated with “secondary bile acid biosynthesis” and negatively correlated with “primary bile acid biosynthesis” in the colonic microbiome of sham-stressed males (Fig. S23 E, F).

In the cecal microbiome of stressed females, “GABAergic synapse” was negatively correlated with uncultured bacterium of *Oscillospiraceae*, *Lachnospiraceae*_NK4A136_group, and *Butyricoccus* and positively correlated with *Christensenellaceae*_R-7_group, *Tyzzerella*, *Oscillospirales*_UCG-010, *Peptococcus*, and *Ruminococcus* (Fig. S24 A, B). “Spinocerebellar ataxia” was strongly associated with *Bacteroides* in both cecal and colonic microbiomes of unstressed sham females and negatively correlated with *Lachnospiraceae*_NK4A136_group in both cecal and colonic microbiomes of stressed sham females (Fig. S24 B, D).

“Ascorbate and aldarate metabolism,” “atrazine degradation,” and “tyrosine metabolism,” upregulated functions in both cecal and colonic microbiomes of stressed GNX females, were positively correlated with uncultured bacterium of *Oscillospiraceae* (Fig. S24 A, C).

“Carbohydrate digestion and absorption,” “bacterial invasion of epithelial cells,” “serotonergic synapse,” and “nonribosomal peptides structures” were strongly associated with *Monoglobus* and Bcilli _RF39 in the colonic microbiome of stressed GNX females and negatively correlated with *Oscillospirales*_UCG-010 in stressed sham females (Fig. S24 E).

## Discussion

The involvement of the gut microbiota in stress regulation is being investigated in a growing number of studies. Several studies in both humans and animals have reported variations in gut microbiota composition depending on the sex of the host [8, 76]. Furthermore, estrogen and testosterone have been shown to directly affect the diversity and composition of the gut microbiome and to be responsible for the sexual dimorphism of gut microbiota [12, 15, 77–79]. In a recent study, using the single prolonged stress, the sex-specific differences in the gut microbial composition and predictive functionality were observed in susceptible and resilient rats [80]. However, the influence of sex and gonadectomy on the response of the gut microbiota to stress remains unexplored. In this study, we investigated the impact of stress on gut microbiota composition and functional pathways in a sex-dependent manner. To account for possible sex-specific differences within the gut microbiota in response to stress, gonadectomy and sham surgery were applied on male and female mice. The microbiome of two parts of the gastrointestinal tract, the colon and the cecum, was analyzed. In addition, the changes in the body weight and the level of corticosterone were evaluated in all mice. The present study shows that stress has a sex-specific effect on the composition of the gut microbiota and functional pathways, and this impact is more obvious in the colon than in the cecum, with males being more affected than females.

All stressed mice displayed a body weight loss and a significant increase in corticosterone levels (Fig. 2). Stress is often associated with weight loss in rodents, and this weight loss is more pronounced in older animals [81–83]. It has also been suggested that weight loss is associated with increased corticosterone and CRH levels in stressed rats [84, 85]. Moreover, stress significantly increase plasma CORT level and bacterial diversity in colonic microbiome of sham animals of both sexes than in their control counterparts and in cecal microbiome of female sham animals than in males **(**Figs. 2, 3**)**. Furthermore, stress did not have any significant effect on all diversity indices in both colonic and cecal microbiomes of gonadectomized mice of both sexes where the main sources of sex hormones are eliminated resulting in the absence or reduced level of sex hormones (Fig. 3). These results indicate that the gut microbiome in sham animals was more susceptible to stress. These findings support the hypothesis that sex hormones may affect gut microbiome diversity [10, 62, 63]. Notably, stress exhibited a sex difference effect on the gut microbiome diversity, with males being significantly affected but not females (Fig. 4A, F, H, Figs. S1, S2 B, E). Likewise, using the mouse model of multi-hit early adversity, males were found to have higher deficits in social behavior and gut microbiota dysbiosis than females [53].

The major effect of stress on bacterial composition was found in the colonic microbiome of sham male mice (Fig. 5, S3, and Tables S2, S3). Interestingly, the abundance of *Lachnospiraceae* (*Lachnospiraceae*_NK4A136_group, *Lachnospiraceae*_UCG-001, and uncultured *Lachnospiraceae* genera) was significantly higher in the gut microbiome of stressed mice of both sexes (Fig. 5). Similarly, *Lachnospiraceae*_NK4A136_group was the common biomarker detected in the gut microbiome of all stressed female mice regardless of surgical status (Fig. S5B, D, E). *Lachnospiraceae*_NK4A136_group was a bacterial marker associated with sham-stressed males (Fig. S4 E, F). Consistent with our data, several other studies have shown an increase in *Lachnospiraceae* in mice exposed to stress [27, 53, 55, 57, 59, 86–89]. Conversely, other authors reported that this bacterial family decreases in stressed animals [30, 54, 90–100]. Although bacterial members of *Lachnospiraceae* are among the largest producers of short-chain fatty acids (mainly butyrate) and are generally considered beneficial for human health, the increased abundance of *Lachnospiraceae* was associated with many metabolic, gastrointestinal, and neurological diseases [101]. The abundance of *Lachnospiraceae* has also been associated with greater weight loss in obese people [102]. The proportion of the bacterial family *Oscillospiraceae* (principally genus *Oscillibacter*) increased in the gut microbiome of all stressed mice (Fig. 5A, C and Tables S2, S3). *Oscillibacter* was determined as a biomarker for the GNX-stressed females compared with GNX controls (Fig. S5D) and for sham-stressed males compared to their control counterpart (Fig. S4E) and compared to the sham-stressed females (Fig. 6D). These data are consistent with results showing that stress and depression are associated with increased abundance of *Oscillibacter* in the gut microbiome of humans and animals [103–110], although a significant decrease of *Oscillibacter* in the gut microbiome of stressed animals has been confirmed by others [95, 111, 112]. Our results showed that the abundance of the bacterial family *Lactobacillaceae* (*Lactobacillus* genus) was decreased in the gut microbiome of all stressed animals (Fig. 5A, C and Tables S2). Similarly, many studies have shown that stress reduced the abundance of *Lactobacillus* in the gut microbiome of rodents, primates,

and humans [55, 56, 89, 94, 96, 100, 104, 109, 113–123] . It has been suggested that the administration of *Lactobacillus* as a probiotic can restore intestinal *Lactobacillus* proportions to improve metabolic alterations and behavioral defects [115]. Remarkably, Rincel et al. [53] have reported that a range of prenatal and early postnatal stresses is associated with an increased abundance of *Lactobacillus* in the gut microbiome of adult males and a decrease in adult females, respectively. Results of our LDA analysis also determined *Lactobacillus* as a biomarker in both the cecum and colon of GNX-stressed males compared to sham-stressed males (Fig. S4C, F). However, LDA analysis of the cecal and colonic microbiome of sham-stressed mice indicated that *Lactobacillus* was the top marker in females compared to males (Fig. 6B, D). It is recommended to consider sex differences in treatment with probiotics in both gut microbiota and behavior disorders [53]. *Bacteroidaceae* family was less abundant in the gut microbiome of sham-stressed mice compared to sham controls, but not in GNX mice (Fig. 5A, C and Tables S2). In addition, *Bacteroides* was significantly correlated with the gut microbiome of sham female control mice (Fig. S5B, E). In accordance with our results, many findings indicated a significant reduction in the relative abundance of the genus *Bacteroides* in humans and rodents exposed to stress [96, 108, 109, 112, 113, 124]. On the other hand, other studies suggested that *Bacteroides* is associated with stress [27, 53, 54, 86, 94, 98, 99, 106, 107, 110, 117, 118, 120, 125–128].

The impact of stress on the gut microbiome composition has a direct effect on the functional pathways of the intestinal microbiota and indirectly on the gut–brain axis in a sex-specific manner. Moreover, the correlation analysis showed that there were multiple correlations between gut bacterial markers and altered functional pathways (Fig. S23, S24). The major dysfunction effect was detected in the colonic microbiome of stressed sham males relative to control sham males, stressed GNX males, and female counterparts (Fig. S17, S18, S20). The difference in functional dysregulation between stressed GNX and sham was significantly higher in males than in females (Fig. S15, S18). In addition, stress-induced CORT levels were significantly higher in GNX males than in sham males (Fig. 2C). Similarly, gonadectomized male rodents showed a significantly higher stress response in comparison with sham males [129, 130]. Findings showed that the lower stress response observed in males injected with testosterone also indicates the dampening effect of testosterone on stress responsiveness [129]. Although the elimination of the main endogenous source of testosterone (orchiectomy) was positively correlated with plasma corticosterone and negatively correlated with gut microbiota dysbiosis in stressed mice, the role of other stress hormones cannot be excluded. For example, other stress hormones such as catecholamines and direct sympathetic modulation of the gut should also be considered as potential modulators of gut microbiome [131]. Our results of functional prediction analysis indicated that stress is associated with profound dysregulation of intestinal functional pathways. Specifically, genes related to membrane transport, energy metabolism, nervous system, neurodegenerative diseases, carbohydrate metabolism, lipid metabolism, amino acid metabolism, and signal transduction mechanisms were upregulated in the gut microbiome of stressed mice. The functional pathways prediction showed that membrane transport pathways (ABC transporters and phosphotransferase system (PTS)), signal transduction pathway (two-component system), and cellular processes (bacterial chemotaxis, flagellar assembly, quorum sensing, and biofilm formation) were enriched in the gut microbiome of stressed mice of both sexes, especially in the colon (Fig. S13, S14, S17, S18, Table S5). The enrichment of these pathways can increase intestinal barrier permeability, known as “leaky gut,” and may disturb signaling between the intestine and the brain [41, 132]. Stress and depression are known to cause gut microbiome dysbiosis and gut barrier hyperpermeability, which consequently results in the disturbance of the gut–brain connection [41, 132]. Pathways of the digestive system (mineral absorption, protein digestion and absorption, fat digestion and absorption, carbohydrate digestion and absorption, cobalamin transport and metabolism, and salivary secretion) were disrupted in both sexes due to stress (Tables S4, S5). In the cecal microbiome of GNX-stressed mice, stress affects the regulation of mineral absorption and cobalamin transport and metabolism in a sex-different manner (Fig. S11, Table S4). The mineral absorption pathway was significantly higher in females, while the cobalamin transport and metabolism pathway was significantly higher in males (Fig. S11, Table S4). Additionally, “cobalamin transport and metabolism” and “protein digestion and absorption” pathways were significantly downregulated in the colonic microbiome of sham-stressed mice of both sexes (Fig. S14, S17, Table S5). Stress may affect the digestion system and reduce intestinal absorption of nutrients [133]. A review study highlighted the involvement of gut microbiome and probiotics in absorbing minerals such as calcium, selenium, zinc, magnesium, and potassium [134]. Probiotics intake may restore intestinal absorption altered by stress [109, 128]. Vitamin B12 (known as cobalamin) can be produced by gut microbiota and plays a crucial role in gut–brain homeostasis [135]. Vitamin B12 deficiency has been associated with an increase in oxidative stress and nervous system impairments [136]. GABAergic synapse pathway was significantly downregulated in the cecal microbiome of both sham- and GNX-stressed females than in their control counterparts (Fig. S6, S7, Table S4) and in the colonic microbiome of sham-stressed males and females than in control mice (Fig. S14, S17, Table S5). Also, GABAergic synapse pathway was significantly lower in the colonic microbiome of sham-stressed males than in sham-stressed females and GNX-stressed males (Fig. S18, S20, Table S5). Gamma-aminobutyric acid (GABA) is an inhibitory neurotransmitter that plays an important role in the central nervous system. Several mechanisms have demonstrated the effects of stress on GABAergic inhibition [137]. Emerging evidence has demonstrated that changes in the levels of GABA are associated with changes in the gut bacterial communities which produce large quantities of GABA [138]. Our results show that downregulation of GABAergic synapse pathway is associated with a higher significant abundance of *Oscillibacter* and *Lachnospiraceae*_NK4A136_group (Fig. S23E). *Oscillibacter* produces valeric acid as its major end product, which is structurally homologous to GABA and able to bind to GABA receptors [103]. The predicted functional analysis of the gut microbiota also revealed the effect of stress on several metabolic pathways that might modulate the host’s metabolic health [139]. We have noted that lipid metabolism was affected by stress in all mice, but crucially in males. It has been reported that acute stress-induced changes in brain lipid metabolites in stress-sensitive Wistar Kyoto rats indicate that lipid metabolism may play a key role in modulating gut–brain signaling in this rat strain [117]. The findings also suggest that lipid metabolism is associated with the imbalanced gut microbiota in major depressive disorder patients [107]. The results of the current study are consistent with reports showing disturbances of lipid metabolism in the gut microbiota of stressed animals [95, 128] and in patients with major depressive disorder [107]. *Lachnospiraceae*_NK4A136_group, uncultured bacterium of *Ruminococcaceae*, and *Butyricicoccaceae*_UCG-009 were positively correlated with “secondary bile acid biosynthesis” and negatively correlated with “primary bile acid biosynthesis” in the colonic microbiome of sham-stressed males (Fig. S23 E, F). Bile acids have now been determined to be a key mediator of the systemic balance between pro- and anti-inflammatory responses in the gut [140]. The bacteria belonging to the *Ruminococcaceae* and *Lachnospiraceae* families are known to produce a 7α-dehydroxylating enzyme that converts primary into secondary bile acids [141]. Findings showed that increased abundance of *Ruminococcaceae* following chronic stress was positively correlated with elevated level of secondary bile acid in the intestine of male mice [142]. “Carbohydrate metabolism” and “glycan biosynthesis and metabolism” pathways were dysregulated in the colonic microbiome of sham-stressed mice of both sexes (Fig. S14, S17, Table S5). “Glycolysis/glycogenesis” pathway was increased in GNX-stressed males compared to the sham-stressed male mice (Fig. S18). Changes in carbohydrate metabolism pathway were associated with gut microbiota disorder in mice and rats under stress [95, 109, 128].

Alterations in carbohydrate metabolism including increased glucose uptake and utilization, hyperlactatemia, increased glucose production, decreased glycogenesis, glucose intolerance, and insulin resistance have been found in patients with stress or trauma [143]. Furthermore, acute stress has been confirmed to affect carbohydrate metabolism through alterations in blood glucose levels and the activity and concentration of various carbohydrate-metabolizing enzymes in rats [144, 145]. A meta-metabolome network of carbohydrate metabolism potentials of the bacterial community in the human gut revealed a total of 27 enzymes exclusively associated with gut bacteria. The majority of these enzymes are present only in *Bacillota* phylum (*Firmicutes*) [146]. Correspondingly, we observed that the enrichment of *Bacillota* in the gut of stressed mice was associated with the enhancement of carbohydrate metabolism. Additionally, *Lactobacillus* was positively correlated with many carbohydrate metabolism pathways including: “C5-branched dibasic acid metabolism,” “glycolysis/gluconeogenesis,” “galactose metabolism,” and “starch and sucrose metabolism” in the gut microbiome of GNX-stressed males (Fig. S23 C, E). Critically, we found that nervous system and neurodegenerative disease pathways were dysregulated in sham-stressed mice (Fig. S11, S14, Table S5). Stress has been shown to be an important factor in promoting the progression of neurodegenerative disorders such as Alzheimer’s, Parkinson’s, and Huntington’s diseases [147].

To our knowledge, the current study represents the first results showing the effect of the interaction of stress and sex on the alteration and dysfunction of the gut microbiome using GNX animals. The results from the present study show not only the profound influence of stress on gut microbiome composition and functional pathways but also strongly demonstrate that many of these changes are sex-dependent. Moreover, a sex-dependent metabolomics analysis might be potentially expanded in order to validate the present results from bacterial predictive functionality and fully exploit the potential sex-dependent activated functions and produced metabolites by the gut microbiota in response to stress. These results lead us to place greater importance on the investigation of how sex might modulate gut–brain axis activity in drug development and pharmacokinetics. Further understanding of the mechanisms of sex-specific regulation of the role of gut microbiota in the stress response may provide interesting preventive, diagnostic, and therapeutic strategies in neurogastroenterology and psychology.

## Supplementary Information

Below is the link to the electronic supplementary material.

ESM 1 (PDF 27.5 MB)

ESM 2 (XLSX 15.6 KB)

ESM 3 (XLSX 27.3 KB)

ESM 4 (XLSX 27.0 KB)

ESM 5 (XLSX 151 KB)

ESM 6 (XLSX 197 KB)

ESM 7 (PDF 651 KB)

## Data Availability

No datasets were generated or analysed during the current study.
