# Supplementary material for: Impact of Sex, Gonadectomy, and Repeated Restraint Stress on Gut Microbiome in Mice
Source: Mol Neurobiol. 2025 Nov 19;63(1):80. doi: 10.1007/s12035-025-05305-6 (PMC12627175; doi:10.1007/s12035-025-05305-6)
Supplement: Supplementary file 1 — (PDF 27.5 MB) [file 12035_2025_5305_MOESM1_ESM.pdf]

Molecular Neurobiology

Impact of sex, gonadectomy and repeated restraint stress on gut microbiome in mice

Chahrazed Mekadim[1*], Jakub Mrázek[1], Martin Vodička[2], Peter Ergang[2], Kateřina Olša Fliegerová[1], Tiziana Maria Mahayri[1], Kallayanee Chawengsaksophak[4], Jiří Pácha[2,3]

[1] Laboratory of Anaerobic Microbiology, Institute of Animal Physiology and Genetics, Czech Academy of Sciences, v.v.i., Prague, Czech Republic.

[2] Laboratory of Epithelial Physiology, Institute of Physiology, Czech Academy of Sciences, v.v.i., Prague, Czech Republic.

[3] Department of Physiology, Faculty of Science, Charles University, Prague, Czech Republic.

[4]Laboratory of Cell Differentiation, Institute of Molecular Genetics, Czech Academy of Sciences, v.v.i., Prague, Czech Republic.

*** Correspondence:**

Chahrazed Mekadim

mekadim@iapg.cas.cz

Fig. S1 Effect of gonadectomy and stress on the beta diversity of the gut microbiome of females. Principal Coordinate Analysis (PCoA) plots based on the Bray-Curtis distance show distinct clusters in the microbiome of cecum (A, B, C) and colon (D, E, F) from different groups of mice. $P \leq 0.05$ was considered statistically significant.

Fig. S2 Effect of gonadectomy and stress on the beta diversity of the gut microbiome of males. Principal Coordinate Analysis (PCoA) plots based on the Bray-Curtis distance show distinct clusters in the microbiome of cecum (A, B, C) and colon (D, E, F) from different groups of mice. $P \leq 0.05$ was considered statistically significant.

Fig. S3 Effect of sex, gonadectomy and stress on the gut microbiota composition. Relative abundance of bacterial populations at phylum level in the microbiome of cecum (A) and colon (B) from different groups of mice.

Fig. S4 Bacterial markers related to stress and gonadectomy in the gut microbiome of males. Linear discriminant analysis effect size (LEfSe) of taxa at genus level in the microbiome of cecum (A, B, C) and colon (D, E, F) from different groups of mice with alpha values of 0.05 and a threshold value of 2.0.

Fig. S5 Bacterial markers related to stress and gonadectomy in the gut microbiome of females. Linear discriminant analysis effect size (LEfSe) of taxa at genus level in the microbiome of cecum (A, B, C) and colon (D, E, F) from different groups of mice with alpha values of 0.05 and a threshold value of 2.0.

Fig. S6 Predicted functional KEGG pathways at level 3 in the cecal microbiome of GNX females.

Fig. S7 Predicted functional KEGG pathways at level 3 in the cecal microbiome of sham females.

Fig. S8 Predicted functional KEGG pathways at level 3 in the cecal microbiome of GNX males.

Fig. S9 Predicted functional KEGG pathways at level 3 in the cecal microbiome of sham males.

Fig. S10 Predicted functional KEGG pathways at level 3 in the cecal microbiome of stressed males.

Fig. S11 Predicted functional KEGG pathways at level 3 in the cecal microbiome of GNX stressed.

Fig. S12 Predicted functional KEGG pathways at level 3 in the cecal microbiome of sham stressed.

Fig. S13 Predicted functional KEGG pathways at level 3 in the colonic microbiome of GNX females.

Fig. S14 Predicted functional KEGG pathways at level 3 in the colonic microbiome of sham females.

Fig. S15 Predicted functional KEGG pathways at level 3 in the colonic microbiome of stressed females.

Fig. S16 Predicted functional KEGG pathways at level 3 in the colonic microbiome of GNX males.

Fig. S17 Predicted functional KEGG pathways at level 3 in the colonic microbiome of sham males.

Fig. S18 Predicted functional KEGG pathways at level 3 in the colonic microbiome of stressed males.

Fig. S19 Predicted functional KEGG pathways at level 3 in the colonic microbiome of GNX stressed.

Fig. S20 Predicted functional KEGG pathways at level 3 in the colonic microbiome of sham stressed.

Fig. S21 Effect of sex, gonadectomy and stress on the gut microbiota composition. Relative abundance of bacterial populations at the family level in the microbiome of cecum (A) and colon (B) from all mice.

Fig. S22 Rarefaction curves. Rarefaction curves of species richness of all samples in cecal microbiome (A) and colonic microbiome (C) and alpha rarefied curves of all samples in cecal microbiome (B) and colonic microbiome (D) based on Shannon index.

Fig. S23 Correlations between identified biomarkers and perturbed functions in the gut microbiome of males. The correlation plot summarizing associations between the bacterial genera markers and altered gut microbiome functions related to stress and gonadectomy in the microbiome of cecum (A, B, C) and colon (D, E, F) of males.

Fig. S24 Correlations between identified biomarkers and perturbed functions in the gut microbiome of females. The correlation plot summarizing associations between the bacterial genera markers and altered gut microbiome functions related to stress and gonadectomy in the microbiome of cecum (A, B) and colon (C,D, E) of females.

Figure S1

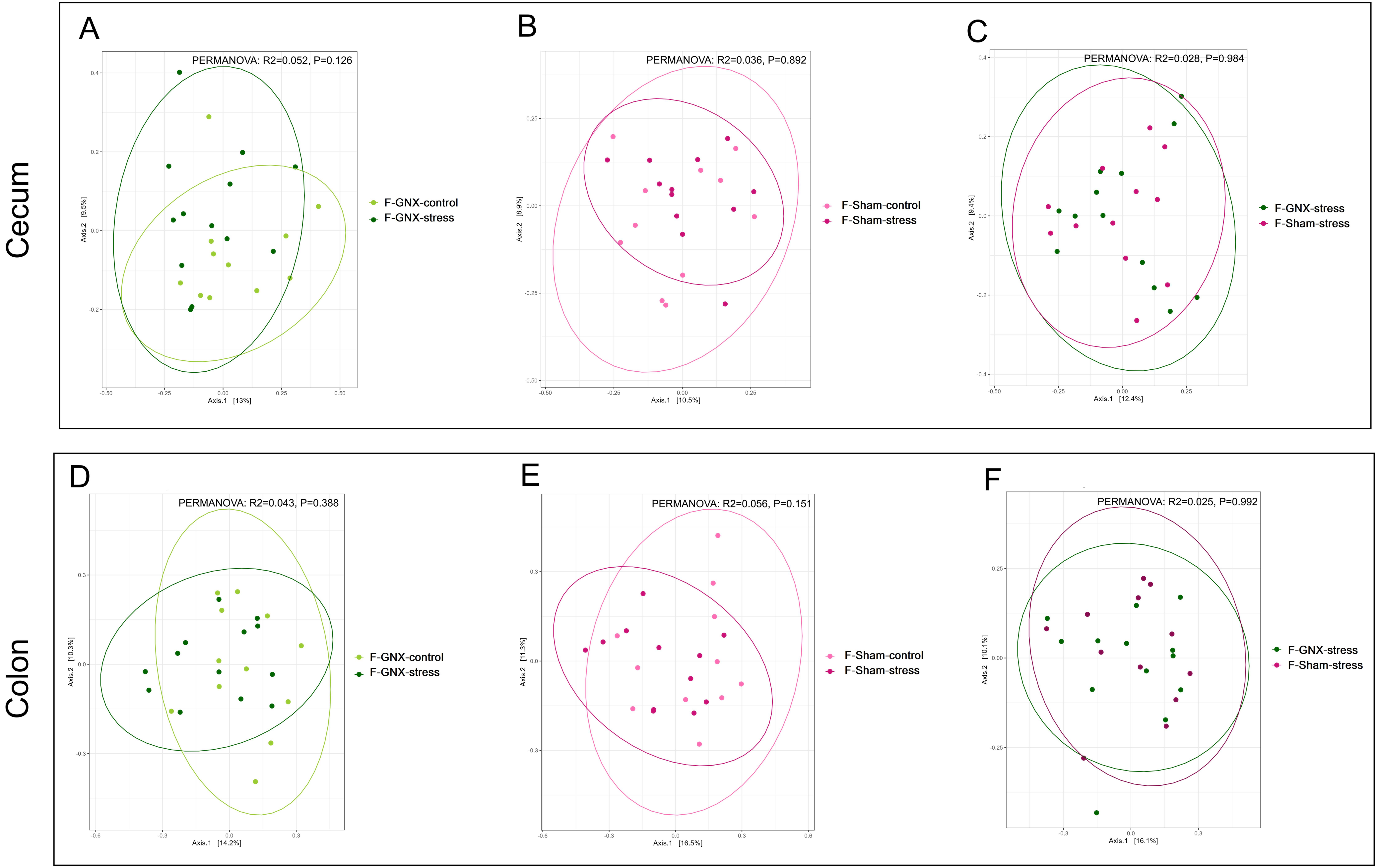

Cecum
A
PERMANOVA: R2=0.052, P=0.126
F-GNX-control
F-GNX-stress
Axis.1 [13%]
Axis.2 [9.5%]
B
PERMANOVA: R2=0.036, P=0.892
F-Sham-control
F-Sham-stress
Axis.1 [10.5%]
Axis.2 [8.9%]
C
PERMANOVA: R2=0.028, P=0.984
F-GNX-stress
F-Sham-stress
Axis.1 [12.4%]
Axis.2 [9.4%]
Colon
D
PERMANOVA: R2=0.043, P=0.388
F-GNX-control
F-GNX-stress
Axis.1 [14.2%]
Axis.2 [10.3%]
E
PERMANOVA: R2=0.056, P=0.151
F-Sham-control
F-Sham-stress
Axis.1 [16.5%]
Axis.2 [11.3%]
F
PERMANOVA: R2=0.025, P=0.992
F-GNX-stress
F-Sham-stress
Axis.1 [16.1%]
Axis.2 [10.1%]

Figure S2

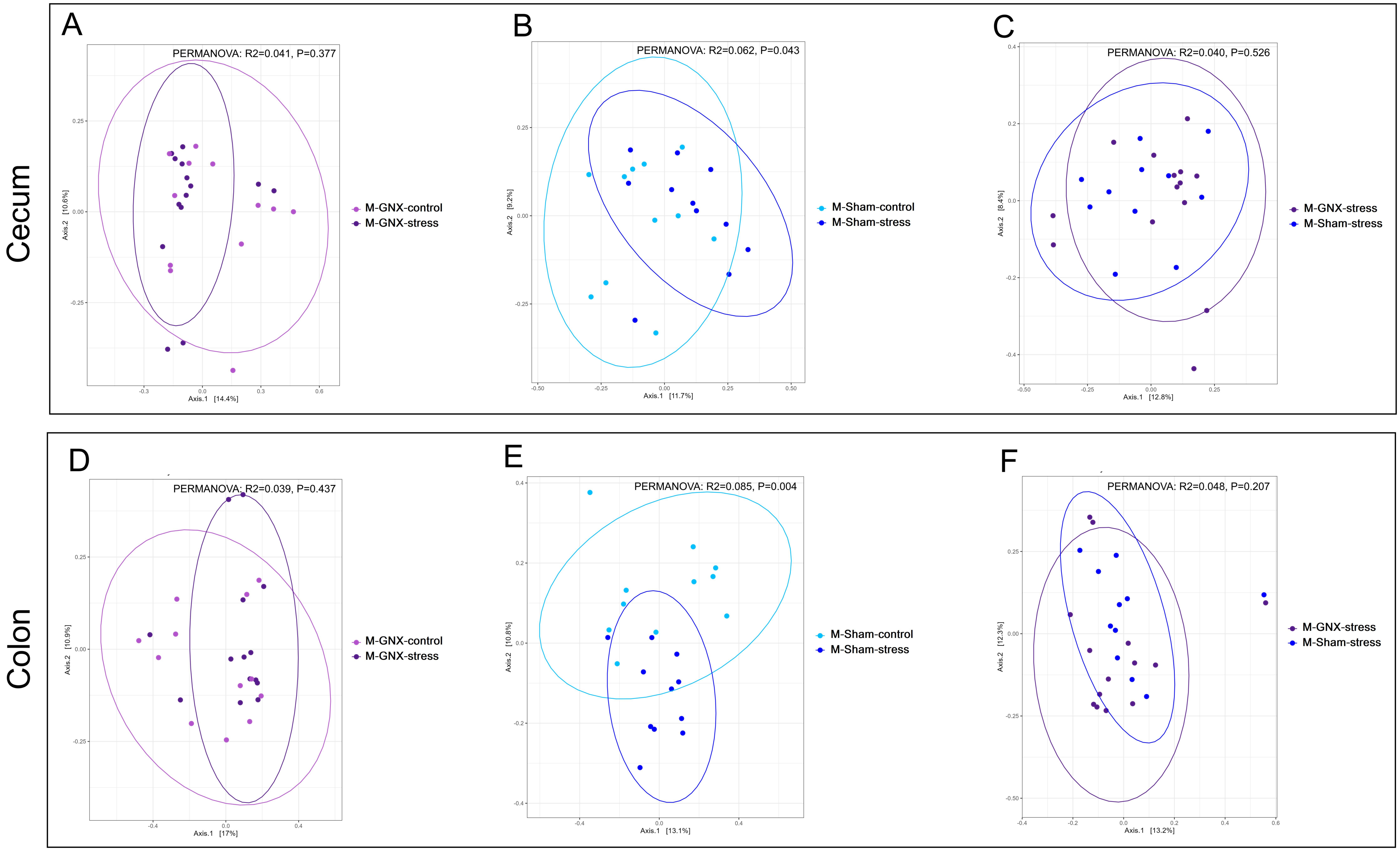
Cecum
Colon
A
PERMANOVA: R2=0.041, P=0.377
Axis.2 [10.6%]
Axis.1 [14.4%]
M-GNX-control
M-GNX-stress
B
PERMANOVA: R2=0.062, P=0.043
Axis.2 [9.2%]
Axis.1 [11.7%]
M-Sham-control
M-Sham-stress
C
PERMANOVA: R2=0.040, P=0.526
Axis.2 [8.4%]
Axis.1 [12.8%]
M-GNX-stress
M-Sham-stress
D
PERMANOVA: R2=0.039, P=0.437
Axis.2 [10.9%]
Axis.1 [17%]
M-GNX-control
M-GNX-stress
E
PERMANOVA: R2=0.085, P=0.004
Axis.2 [10.8%]
Axis.1 [13.1%]
M-Sham-control
M-Sham-stress
F
PERMANOVA: R2=0.048, P=0.207
Axis.2 [12.3%]
Axis.1 [13.2%]
M-GNX-stress
M-Sham-stress

Figure S3

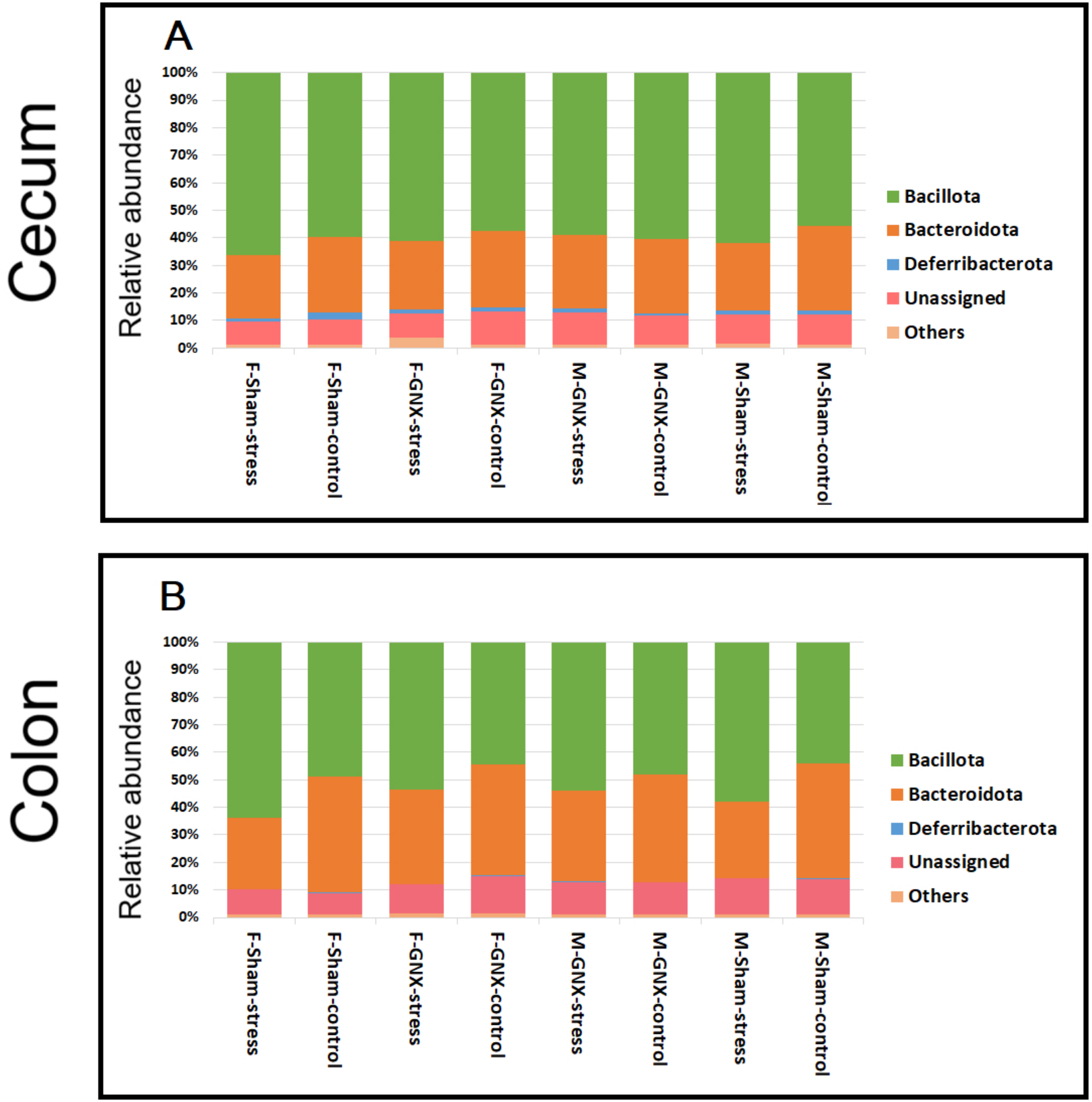
Cecum
A
Relative abundance
100%
90%
80%
70%
60%
50%
40%
30%
20%
10%
0%
F-Sham-stress
F-Sham-control
F-GNX-stress
F-GNX-control
M-GNX-stress
M-GNX-control
M-Sham-stress
M-Sham-control
Bacillota
Bacteroidota
Deferribacterota
Unassigned
Others
Colon
B
Relative abundance
100%
90%
80%
70%
60%
50%
40%
30%
20%
10%
0%
F-Sham-stress
F-Sham-control
F-GNX-stress
F-GNX-control
M-GNX-stress
M-GNX-control
M-Sham-stress
M-Sham-control
Bacillota
Bacteroidota
Deferribacterota
Unassigned
Others

Figure S4

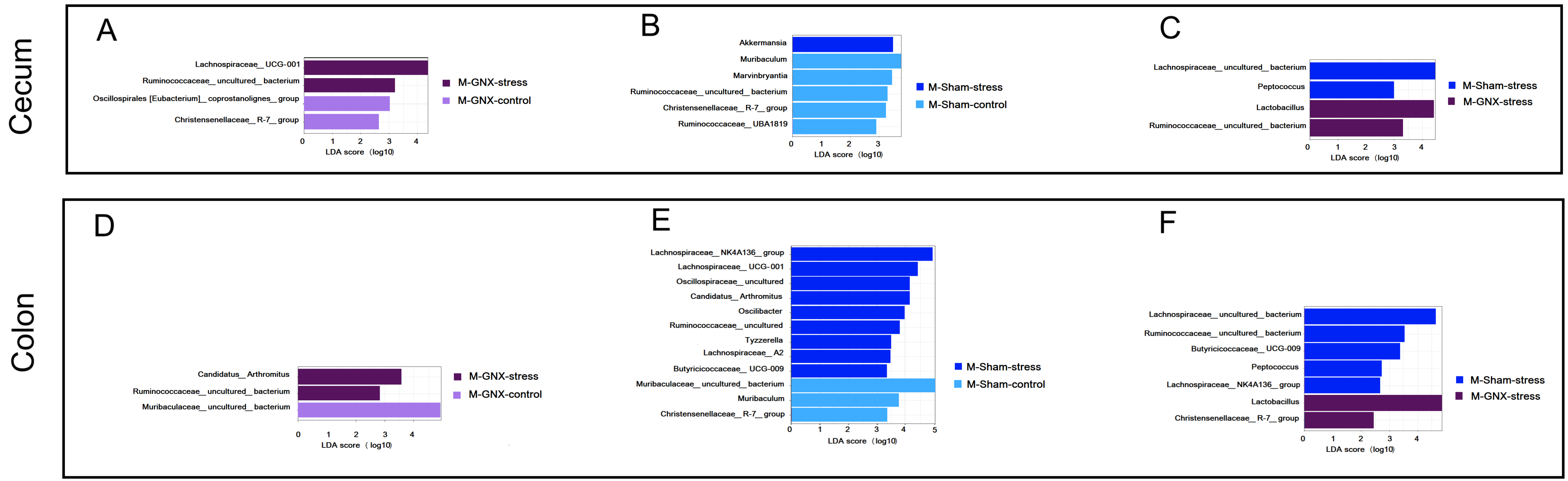
Cecum
A
Lachnospiraceae_UCG-001
Ruminococcaceae_uncultured_bacterium
Oscillospirales [Eubacterium]_coprostanolignes_group
Christensenellaceae_R-7_group
LDA score (log10)
M-GNX-stress
M-GNX-control
B
Akkermansia
Muribaculum
Marvinbryantia
Ruminococcaceae_uncultured_bacterium
Christensenellaceae_R-7_group
Ruminococcaceae_UBA1819
LDA score (log10)
M-Sham-stress
M-Sham-control
C
Lachnospiraceae_uncultured_bacterium
Peptococcus
Lactobacillus
Ruminococcaceae_uncultured_bacterium
LDA score (log10)
M-Sham-stress
M-GNX-stress
Colon
D
Candidatus_Arthromitus
Ruminococcaceae_uncultured_bacterium
Muribaculaceae_uncultured_bacterium
LDA score (log10)
M-GNX-stress
M-GNX-control
E
Lachnospiraceae_NK4A136_group
Lachnospiraceae_UCG-001
Oscillospiraceae_uncultured
Candidatus_Arthromitus
Oscilibacter
Ruminococcaceae_uncultured
Tyzzerella
Lachnospiraceae_A2
Butyricicoccaceae_UCG-009
Muribaculaceae_uncultured_bacterium
Muribaculum
Christensenellaceae_R-7_group
LDA score (log10)
M-Sham-stress
M-Sham-control
F
Lachnospiraceae_uncultured_bacterium
Ruminococcaceae_uncultured_bacterium
Butyricicoccaceae_UCG-009
Peptococcus
Lachnospiraceae_NK4A136_group
Lactobacillus
Christensenellaceae_R-7_group
LDA score (log10)
M-Sham-stress
M-GNX-stress

Figure S5

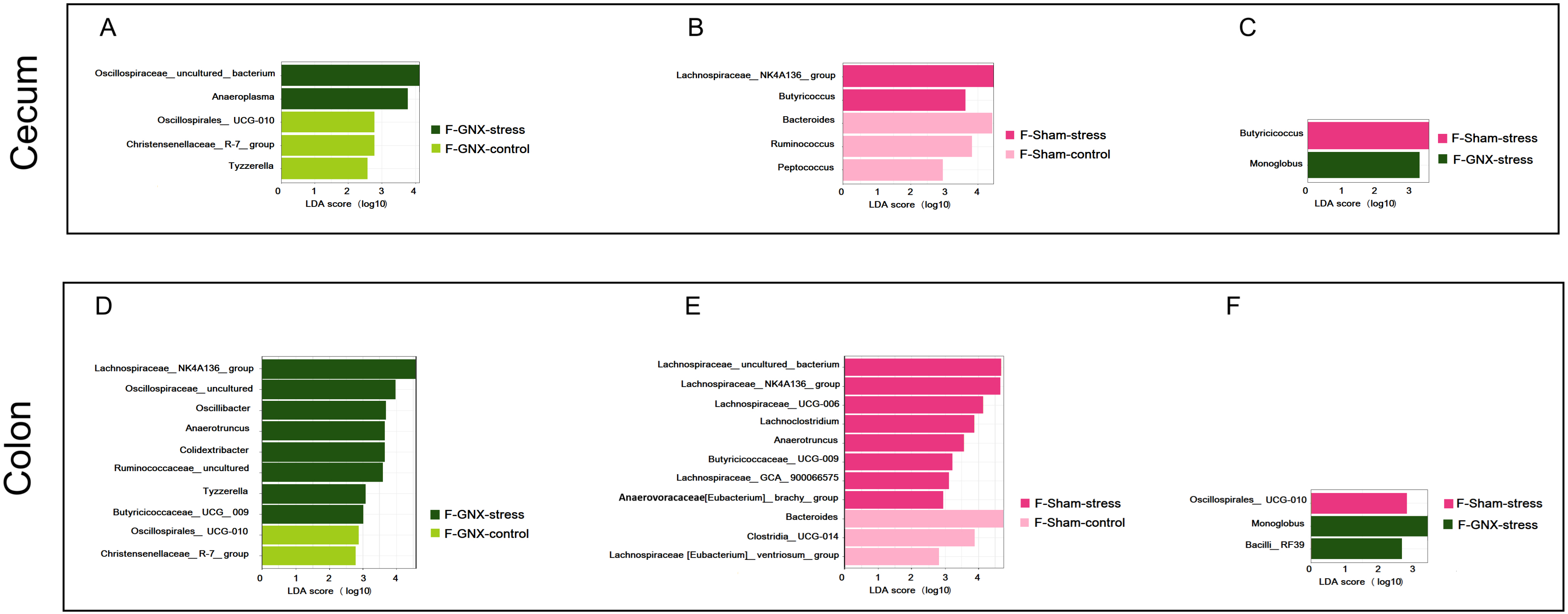

Cecum
A
Oscillospiraceae_ uncultured_ bacterium
Anaeroplasma
Oscillospirales_ UCG-010
Christensenellaceae_ R-7_ group
Tyzzerella
F-GNX-stress
F-GNX-control
LDA score (log10)
B
Lachnospiraceae_ NK4A136_ group
Butyricoccus
Bacteroides
Ruminococcus
Peptococcus
F-Sham-stress
F-Sham-control
LDA score (log10)
C
Butyricicoccus
Monoglobus
F-Sham-stress
F-GNX-stress
LDA score (log10)
Colon
D
Lachnospiraceae_ NK4A136_ group
Oscillospiraceae_ uncultured
Oscillibacter
Anaerotruncus
Colidextribacter
Ruminococcaceae_ uncultured
Tyzzerella
Butyricicoccaceae_ UCG_ 009
Oscillospirales_ UCG-010
Christensenellaceae_ R-7_ group
F-GNX-stress
F-GNX-control
LDA score (log10)
E
Lachnospiraceae_ uncultured_ bacterium
Lachnospiraceae_ NK4A136_ group
Lachnospiraceae_ UCG-006
Lachnoclostridium
Anaerotruncus
Butyricicoccaceae_ UCG-009
Lachnospiraceae_ GCA_ 900066575
Anaerovoracaceae[Eubacterium]_ brachy_ group
Bacteroides
Clostridia_ UCG-014
Lachnospiraceae [Eubacterium]_ ventriosum_ group
F-Sham-stress
F-Sham-control
LDA score (log10)
F
Oscillospirales_ UCG-010
Monoglobus
Bacilli_ RF39
F-Sham-stress
F-GNX-stress
LDA score (log10)

Figure S6

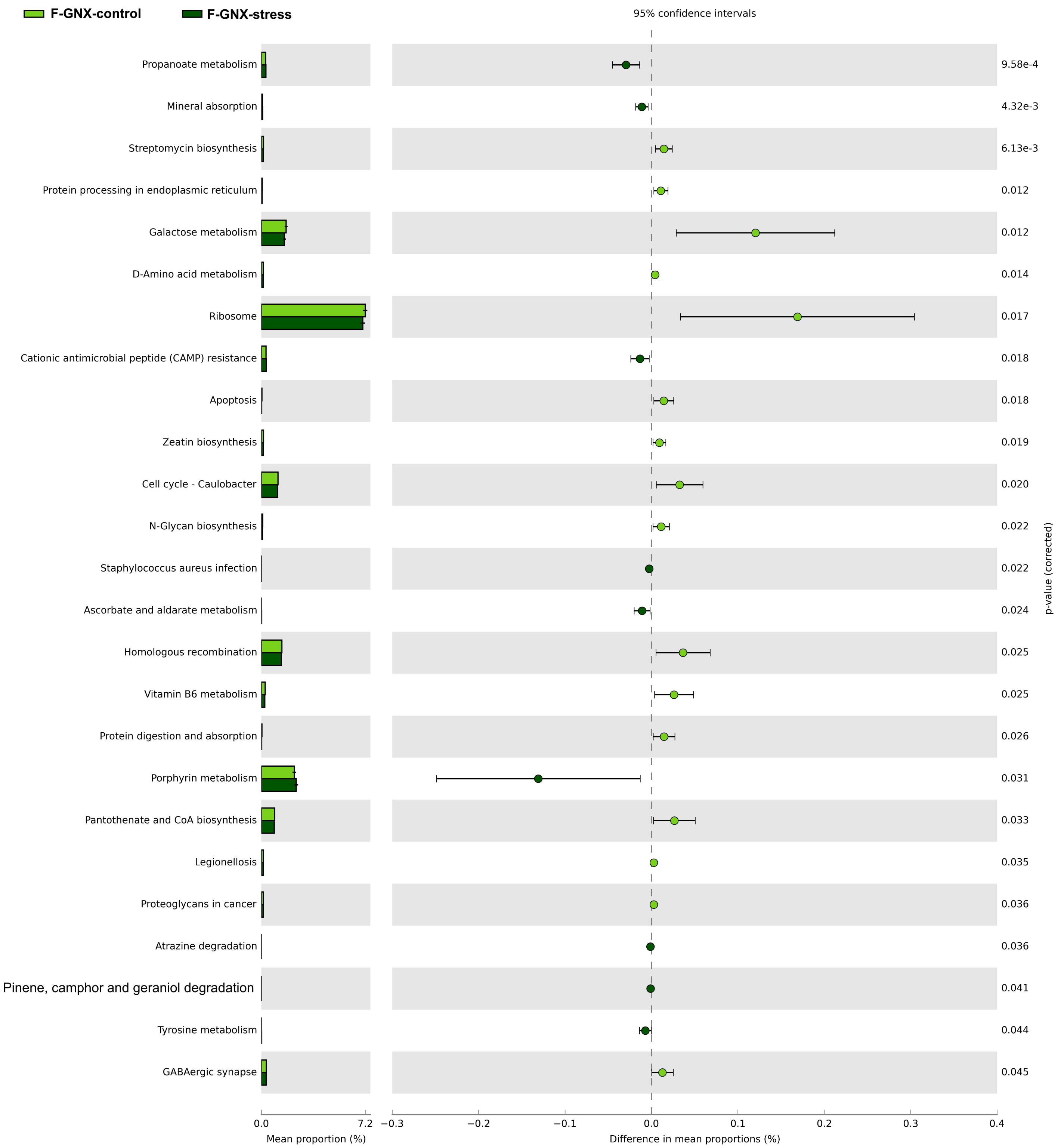

F-GNX-control
F-GNX-stress
95% confidence intervals
Propanoate metabolism
Mineral absorption
Streptomycin biosynthesis
Protein processing in endoplasmic reticulum
Galactose metabolism
D-Amino acid metabolism
Ribosome
Cationic antimicrobial peptide (CAMP) resistance
Apoptosis
Zeatin biosynthesis
Cell cycle - Caulobacter
N-Glycan biosynthesis
Staphylococcus aureus infection
Ascorbate and aldarate metabolism
Homologous recombination
Vitamin B6 metabolism
Protein digestion and absorption
Porphyrin metabolism
Pantothenate and CoA biosynthesis
Legionellosis
Proteoglycans in cancer
Atrazine degradation
Pinene, camphor and geraniol degradation
Tyrosine metabolism
GABAergic synapse
9.58e-4
4.32e-3
6.13e-3
0.012
0.012
0.014
0.017
0.018
0.018
0.019
0.020
0.022
0.022
0.024
0.025
0.025
0.026
0.031
0.033
0.035
0.036
0.036
0.041
0.044
0.045
p-value (corrected)
0.0
7.2
Mean proportion (%)
−0.3
−0.2
−0.1
0.0
0.1
0.2
0.3
0.4
Difference in mean proportions (%)

Figure S7

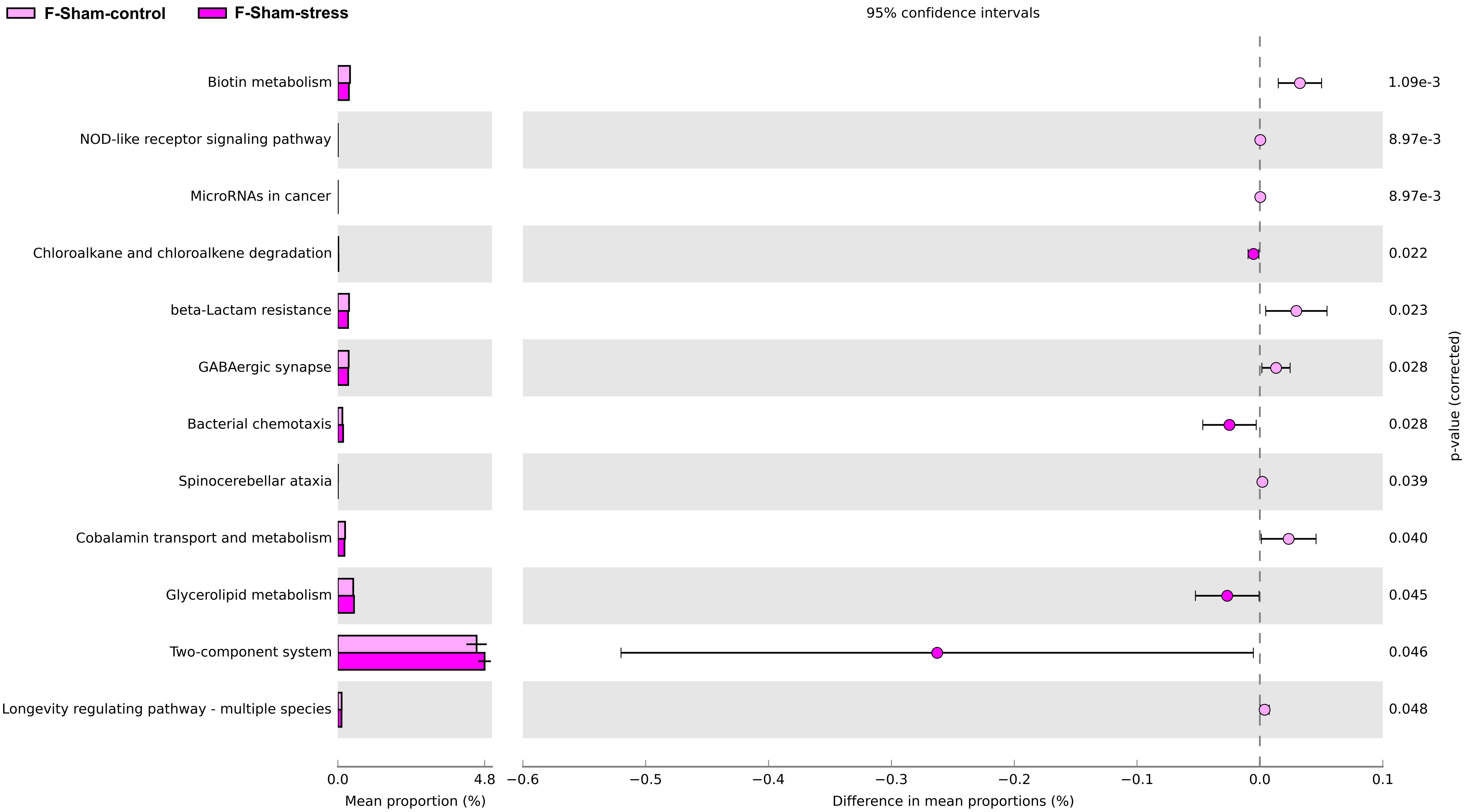

F-Sham-control
F-Sham-stress
95% confidence intervals
Biotin metabolism
NOD-like receptor signaling pathway
MicroRNAs in cancer
Chloroalkane and chloroalkene degradation
beta-Lactam resistance
GABAergic synapse
Bacterial chemotaxis
Spinocerebellar ataxia
Cobalamin transport and metabolism
Glycerolipid metabolism
Two-component system
Longevity regulating pathway - multiple species
1.09e-3
8.97e-3
8.97e-3
0.022
0.023
0.028
0.028
0.039
0.040
0.045
0.046
0.048
p-value (corrected)
0.0
4.8
Mean proportion (%)
−0.6
−0.5
−0.4
−0.3
−0.2
−0.1
0.0
0.1
Difference in mean proportions (%)

Figure S8

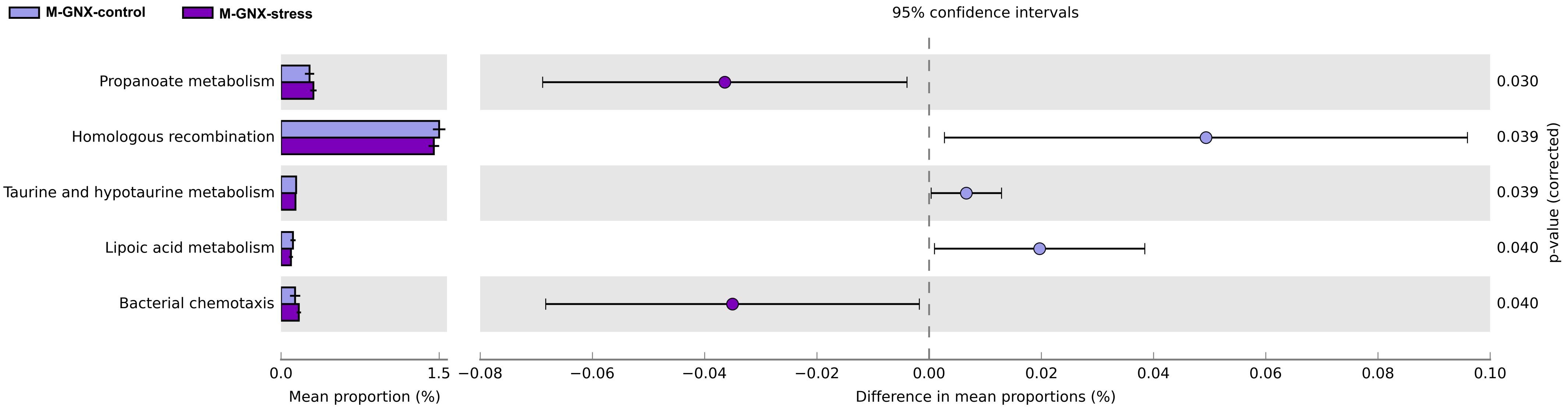

M-GNX-control
M-GNX-stress
95% confidence intervals
Propanoate metabolism
Homologous recombination
Taurine and hypotaurine metabolism
Lipoic acid metabolism
Bacterial chemotaxis
0.030
0.039
0.039
0.040
0.040
p-value (corrected)
0.0
1.5
Mean proportion (%)
−0.08
−0.06
−0.04
−0.02
0.00
0.02
0.04
0.06
0.08
0.10
Difference in mean proportions (%)

Figure S9

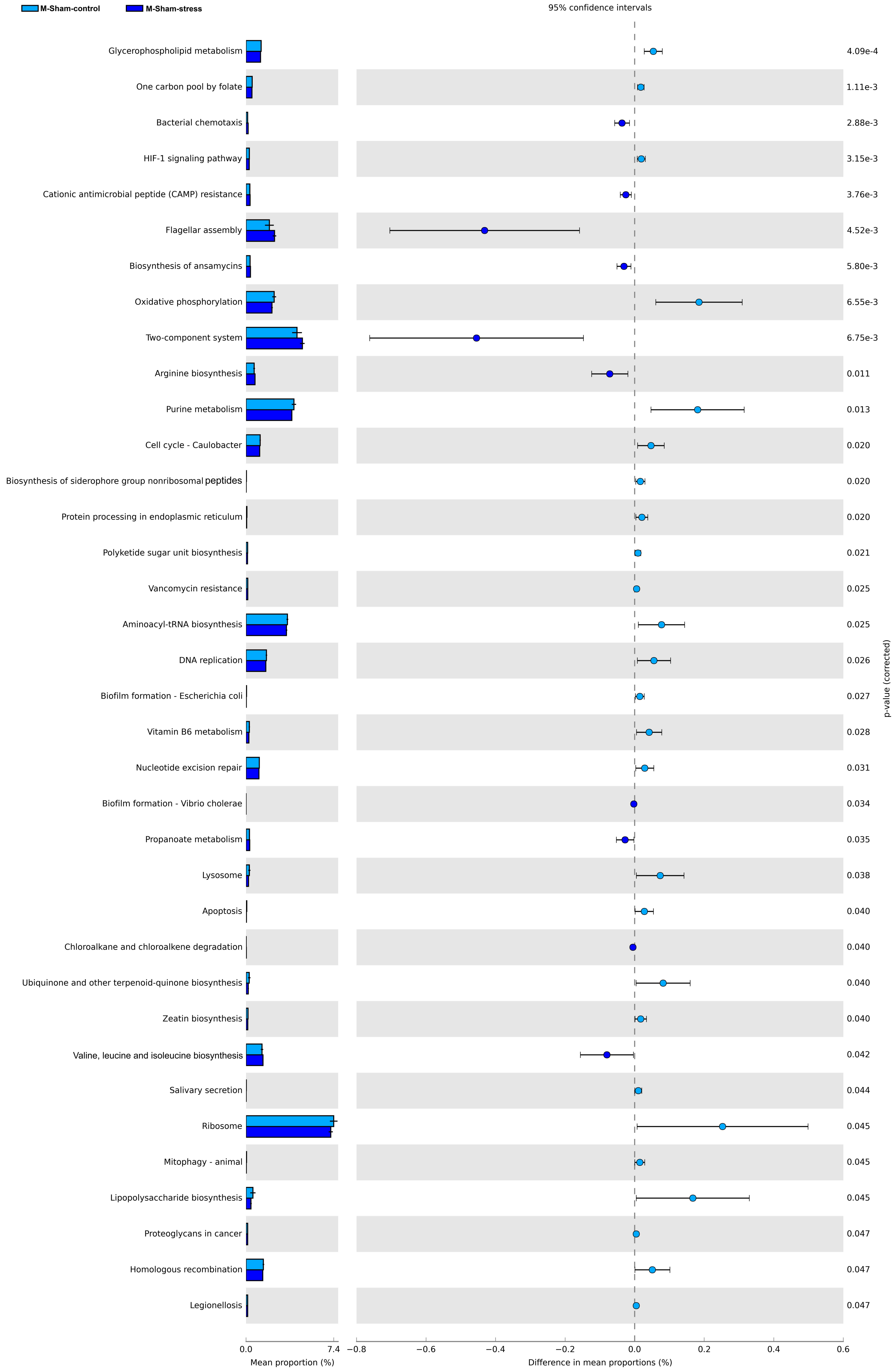

M-Sham-control
M-Sham-stress
95% confidence intervals
Glycerophospholipid metabolism
One carbon pool by folate
Bacterial chemotaxis
HIF-1 signaling pathway
Cationic antimicrobial peptide (CAMP) resistance
Flagellar assembly
Biosynthesis of ansamycins
Oxidative phosphorylation
Two-component system
Arginine biosynthesis
Purine metabolism
Cell cycle - Caulobacter
Biosynthesis of siderophore group nonribosomal peptides
Protein processing in endoplasmic reticulum
Polyketide sugar unit biosynthesis
Vancomycin resistance
Aminoacyl-tRNA biosynthesis
DNA replication
Biofilm formation - Escherichia coli
Vitamin B6 metabolism
Nucleotide excision repair
Biofilm formation - Vibrio cholerae
Propanoate metabolism
Lysosome
Apoptosis
Chloroalkane and chloroalkene degradation
Ubiquinone and other terpenoid-quinone biosynthesis
Zeatin biosynthesis
Valine, leucine and isoleucine biosynthesis
Salivary secretion
Ribosome
Mitophagy - animal
Lipopolysaccharide biosynthesis
Proteoglycans in cancer
Homologous recombination
Legionellosis
4.09e-4
1.11e-3
2.88e-3
3.15e-3
3.76e-3
4.52e-3
5.80e-3
6.55e-3
6.75e-3
0.011
0.013
0.020
0.020
0.020
0.021
0.025
0.025
0.026
0.027
0.028
0.031
0.034
0.035
0.038
0.040
0.040
0.040
0.040
0.042
0.044
0.045
0.045
0.045
0.047
0.047
0.047
p-value (corrected)
0.0
7.4
Mean proportion (%)
−0.8
−0.6
−0.4
−0.2
0.0
0.2
0.4
0.6
Difference in mean proportions (%)

Figure S10

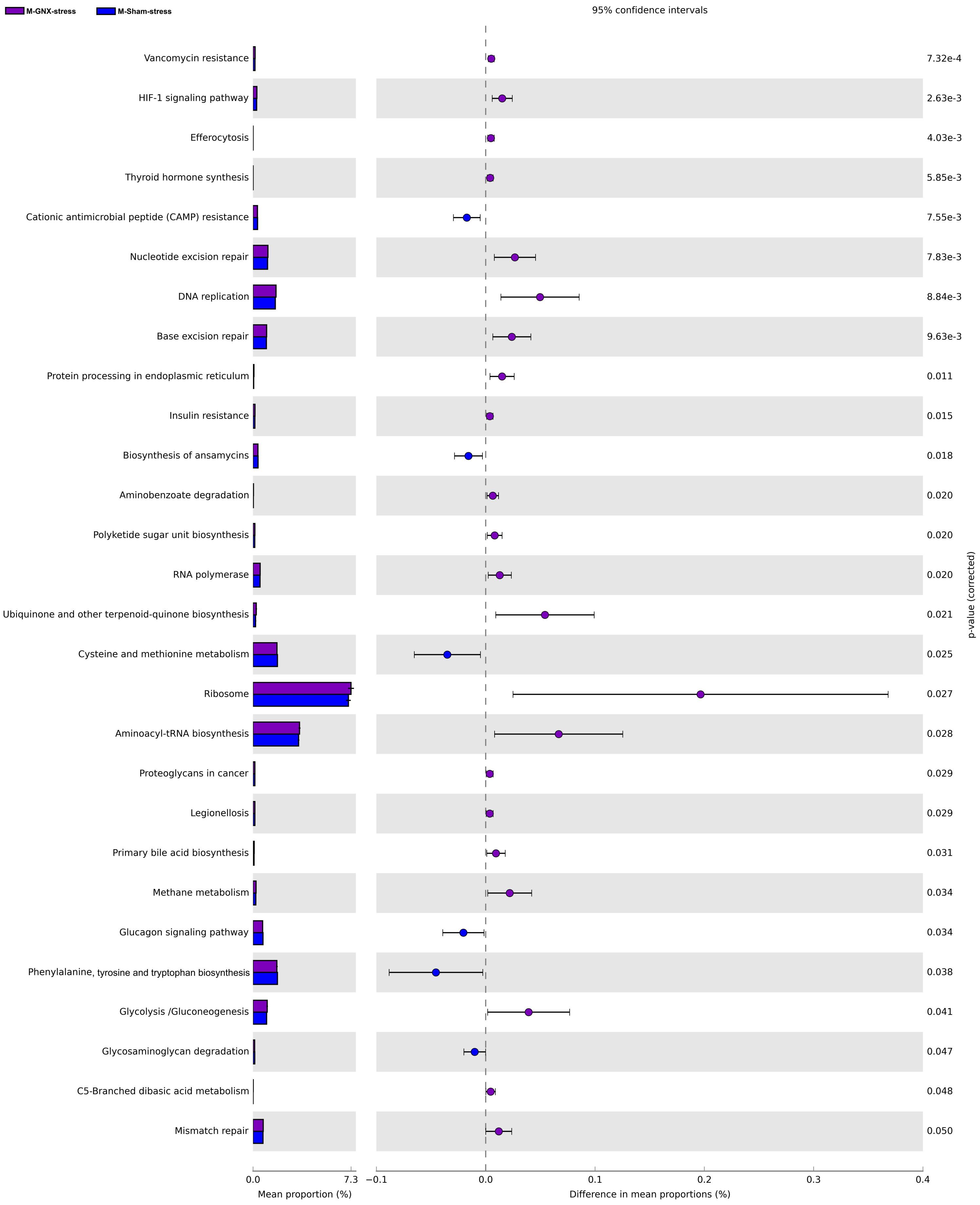

M-GNX-stress
M-Sham-stress
95% confidence intervals
Vancomycin resistance
HIF-1 signaling pathway
Efferocytosis
Thyroid hormone synthesis
Cationic antimicrobial peptide (CAMP) resistance
Nucleotide excision repair
DNA replication
Base excision repair
Protein processing in endoplasmic reticulum
Insulin resistance
Biosynthesis of ansamycins
Aminobenzoate degradation
Polyketide sugar unit biosynthesis
RNA polymerase
Ubiquinone and other terpenoid-quinone biosynthesis
Cysteine and methionine metabolism
Ribosome
Aminoacyl-tRNA biosynthesis
Proteoglycans in cancer
Legionellosis
Primary bile acid biosynthesis
Methane metabolism
Glucagon signaling pathway
Phenylalanine, tyrosine and tryptophan biosynthesis
Glycolysis /Gluconeogenesis
Glycosaminoglycan degradation
C5-Branched dibasic acid metabolism
Mismatch repair
7.32e-4
2.63e-3
4.03e-3
5.85e-3
7.55e-3
7.83e-3
8.84e-3
9.63e-3
0.011
0.015
0.018
0.020
0.020
0.020
0.021
0.025
0.027
0.028
0.029
0.029
0.031
0.034
0.034
0.038
0.041
0.047
0.048
0.050
p-value (corrected)
0.0
7.3
Mean proportion (%)
−0.1
0.0
0.1
0.2
0.3
0.4
Difference in mean proportions (%)

Figure S11

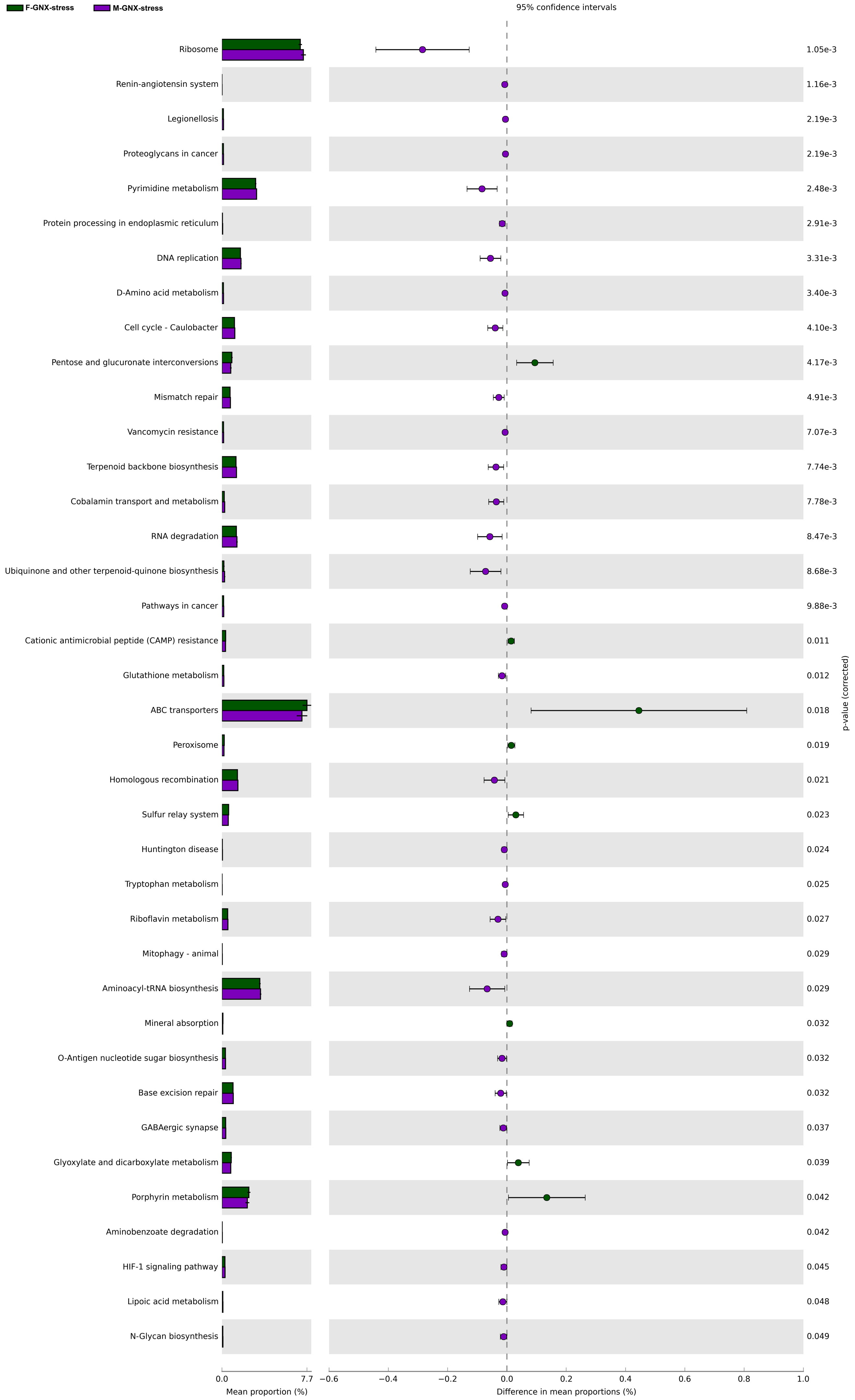

F-GNX-stress
M-GNX-stress
95% confidence intervals
Ribosome
Renin-angiotensin system
Legionellosis
Proteoglycans in cancer
Pyrimidine metabolism
Protein processing in endoplasmic reticulum
DNA replication
D-Amino acid metabolism
Cell cycle - Caulobacter
Pentose and glucuronate interconversions
Mismatch repair
Vancomycin resistance
Terpenoid backbone biosynthesis
Cobalamin transport and metabolism
RNA degradation
Ubiquinone and other terpenoid-quinone biosynthesis
Pathways in cancer
Cationic antimicrobial peptide (CAMP) resistance
Glutathione metabolism
ABC transporters
Peroxisome
Homologous recombination
Sulfur relay system
Huntington disease
Tryptophan metabolism
Riboflavin metabolism
Mitophagy - animal
Aminoacyl-tRNA biosynthesis
Mineral absorption
O-Antigen nucleotide sugar biosynthesis
Base excision repair
GABAergic synapse
Glyoxylate and dicarboxylate metabolism
Porphyrin metabolism
Aminobenzoate degradation
HIF-1 signaling pathway
Lipoic acid metabolism
N-Glycan biosynthesis
1.05e-3
1.16e-3
2.19e-3
2.19e-3
2.48e-3
2.91e-3
3.31e-3
3.40e-3
4.10e-3
4.17e-3
4.91e-3
7.07e-3
7.74e-3
7.78e-3
8.47e-3
8.68e-3
9.88e-3
0.011
0.012
0.018
0.019
0.021
0.023
0.024
0.025
0.027
0.029
0.029
0.032
0.032
0.032
0.037
0.039
0.042
0.042
0.045
0.048
0.049
p-value (corrected)
0.0
7.7
Mean proportion (%)
−0.6
−0.4
−0.2
0.0
0.2
0.4
0.6
0.8
1.0
Difference in mean proportions (%)

Figure S12

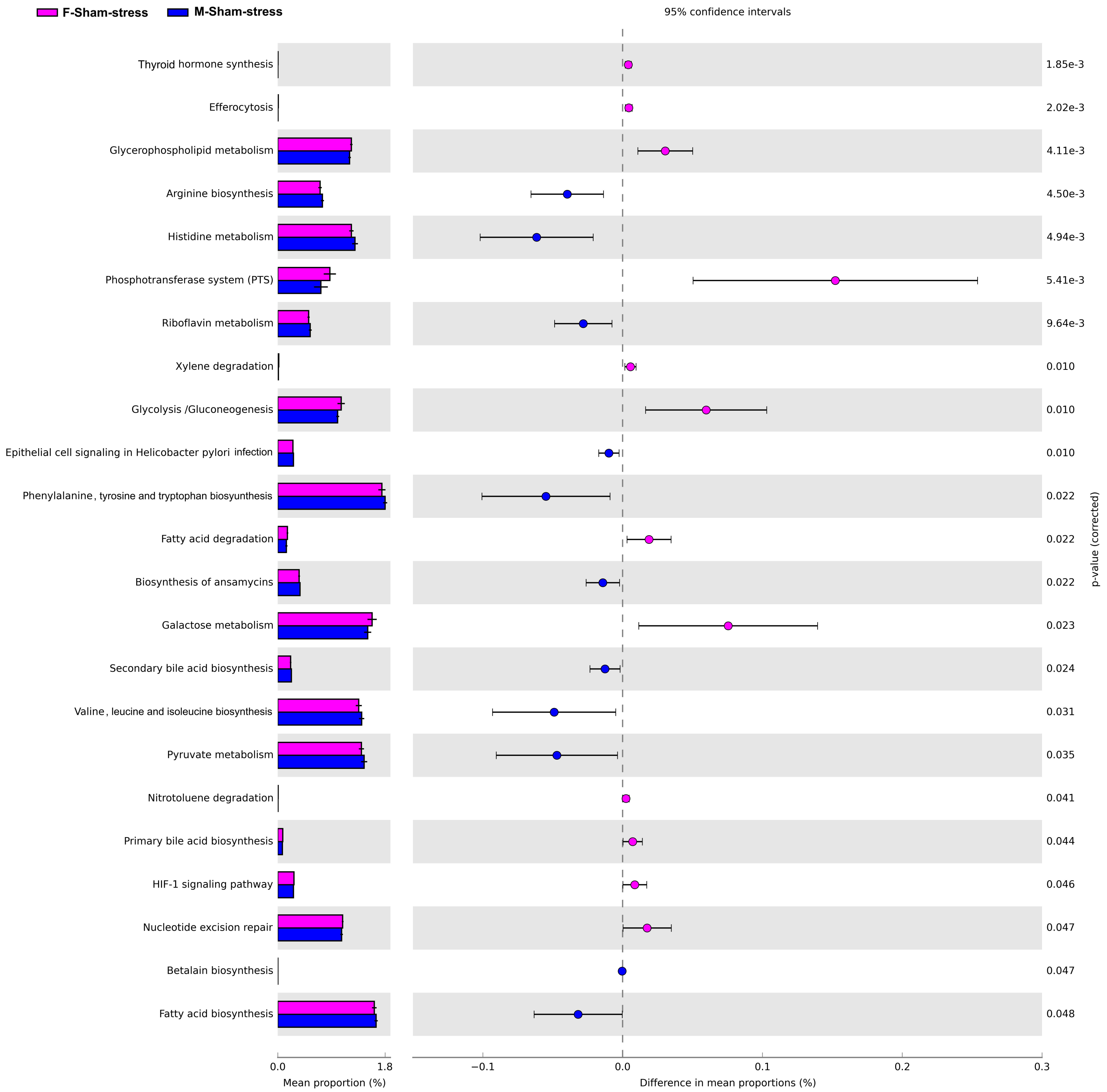

F-Sham-stress
M-Sham-stress
95% confidence intervals
Thyroid hormone synthesis
Efferocytosis
Glycerophospholipid metabolism
Arginine biosynthesis
Histidine metabolism
Phosphotransferase system (PTS)
Riboflavin metabolism
Xylene degradation
Glycolysis /Gluconeogenesis
Epithelial cell signaling in Helicobacter pylori infection
Phenylalanine, tyrosine and tryptophan biosynthesis
Fatty acid degradation
Biosynthesis of ansamycins
Galactose metabolism
Secondary bile acid biosynthesis
Valine, leucine and isoleucine biosynthesis
Pyruvate metabolism
Nitrotoluene degradation
Primary bile acid biosynthesis
HIF-1 signaling pathway
Nucleotide excision repair
Betalain biosynthesis
Fatty acid biosynthesis
1.85e-3
2.02e-3
4.11e-3
4.50e-3
4.94e-3
5.41e-3
9.64e-3
0.010
0.010
0.010
0.022
0.022
0.022
0.023
0.024
0.031
0.035
0.041
0.044
0.046
0.047
0.047
0.048
p-value (corrected)
0.0
1.8
Mean proportion (%)
−0.1
0.0
0.1
0.2
0.3
Difference in mean proportions (%)

Figure S13

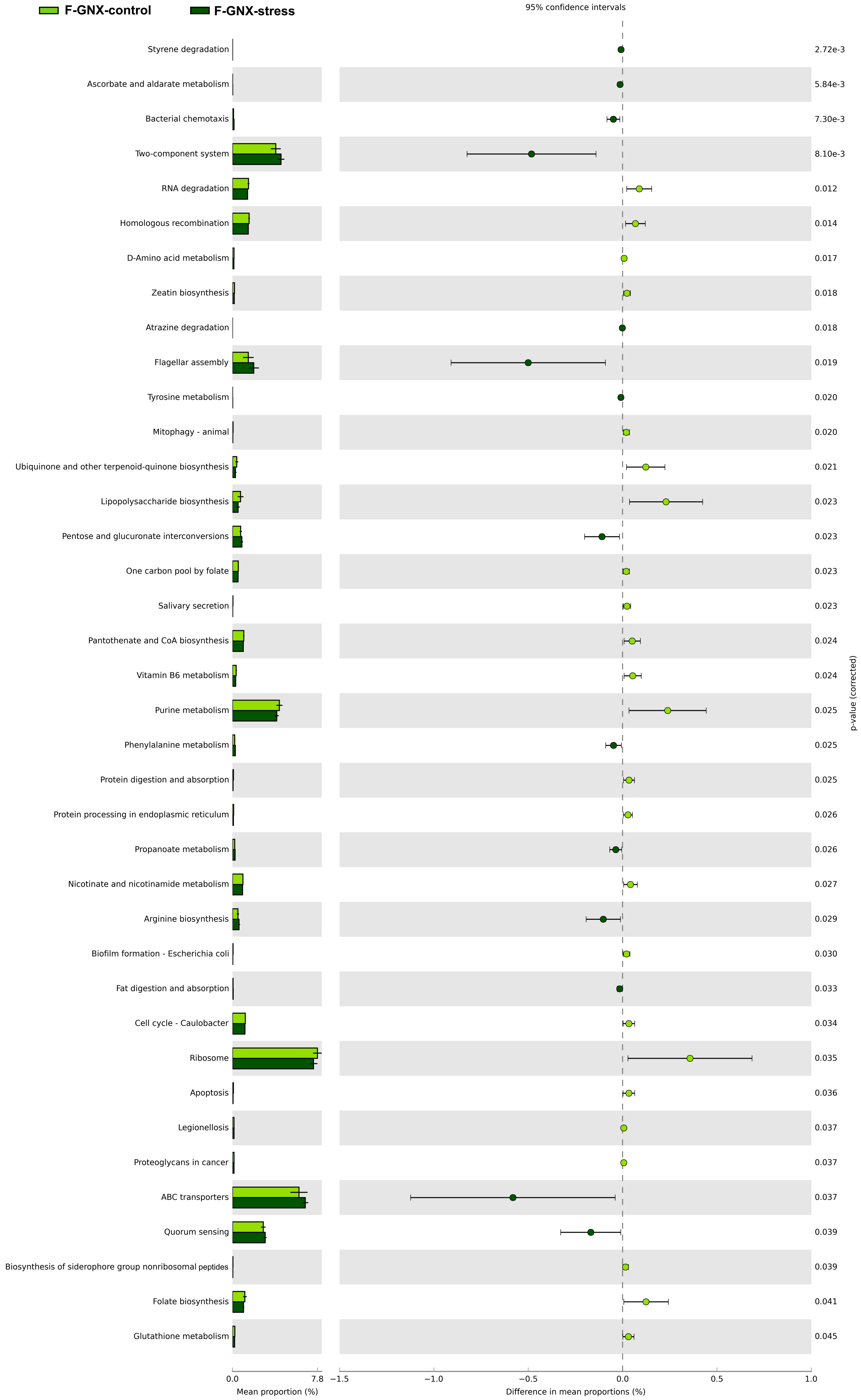

F-GNX-control
F-GNX-stress
95% confidence intervals
Styrene degradation
Ascorbate and aldarate metabolism
Bacterial chemotaxis
Two-component system
RNA degradation
Homologous recombination
D-Amino acid metabolism
Zeatin biosynthesis
Atrazine degradation
Flagellar assembly
Tyrosine metabolism
Mitophagy - animal
Ubiquinone and other terpenoid-quinone biosynthesis
Lipopolysaccharide biosynthesis
Pentose and glucuronate interconversions
One carbon pool by folate
Salivary secretion
Pantothenate and CoA biosynthesis
Vitamin B6 metabolism
Purine metabolism
Phenylalanine metabolism
Protein digestion and absorption
Protein processing in endoplasmic reticulum
Propanoate metabolism
Nicotinate and nicotinamide metabolism
Arginine biosynthesis
Biofilm formation - Escherichia coli
Fat digestion and absorption
Cell cycle - Caulobacter
Ribosome
Apoptosis
Legionellosis
Proteoglycans in cancer
ABC transporters
Quorum sensing
Biosynthesis of siderophore group nonribosomal peptides
Folate biosynthesis
Glutathione metabolism
2.72e-3
5.84e-3
7.30e-3
8.10e-3
0.012
0.014
0.017
0.018
0.018
0.019
0.020
0.020
0.021
0.023
0.023
0.023
0.023
0.024
0.024
0.025
0.025
0.025
0.026
0.026
0.027
0.029
0.030
0.033
0.034
0.035
0.036
0.037
0.037
0.037
0.039
0.039
0.041
0.045
p-value (corrected)
0.0
7.8
Mean proportion (%)
−1.5
−1.0
−0.5
0.0
0.5
1.0
Difference in mean proportions (%)

Figure S14

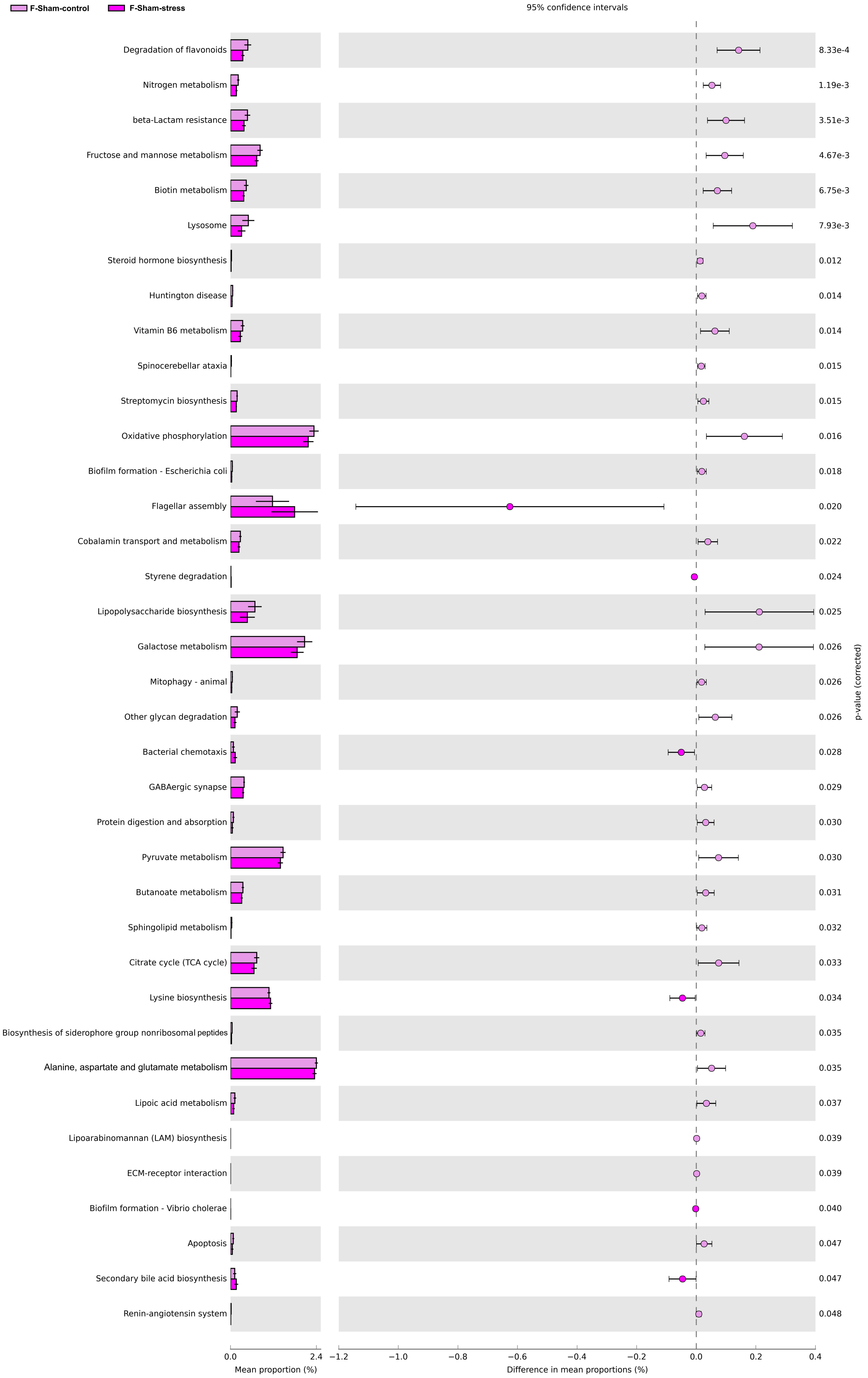
F-Sham-control
F-Sham-stress
95% confidence intervals
Degradation of flavonoids
Nitrogen metabolism
beta-Lactam resistance
Fructose and mannose metabolism
Biotin metabolism
Lysosome
Steroid hormone biosynthesis
Huntington disease
Vitamin B6 metabolism
Spinocerebellar ataxia
Streptomycin biosynthesis
Oxidative phosphorylation
Biofilm formation - Escherichia coli
Flagellar assembly
Cobalamin transport and metabolism
Styrene degradation
Lipopolysaccharide biosynthesis
Galactose metabolism
Mitophagy - animal
Other glycan degradation
Bacterial chemotaxis
GABAergic synapse
Protein digestion and absorption
Pyruvate metabolism
Butanoate metabolism
Sphingolipid metabolism
Citrate cycle (TCA cycle)
Lysine biosynthesis
Biosynthesis of siderophore group nonribosomal peptides
Alanine, aspartate and glutamate metabolism
Lipoic acid metabolism
Lipoarabinomannan (LAM) biosynthesis
ECM-receptor interaction
Biofilm formation - Vibrio cholerae
Apoptosis
Secondary bile acid biosynthesis
Renin-angiotensin system
8.33e-4
1.19e-3
3.51e-3
4.67e-3
6.75e-3
7.93e-3
0.012
0.014
0.014
0.015
0.015
0.016
0.018
0.020
0.022
0.024
0.025
0.026
0.026
0.026
0.028
0.029
0.030
0.030
0.031
0.032
0.033
0.034
0.035
0.035
0.037
0.039
0.039
0.040
0.047
0.047
0.048
p-value (corrected)
0.0
2.4
Mean proportion (%)
−1.2
−1.0
−0.8
−0.6
−0.4
−0.2
0.0
0.2
0.4
Difference in mean proportions (%)

Figure S15

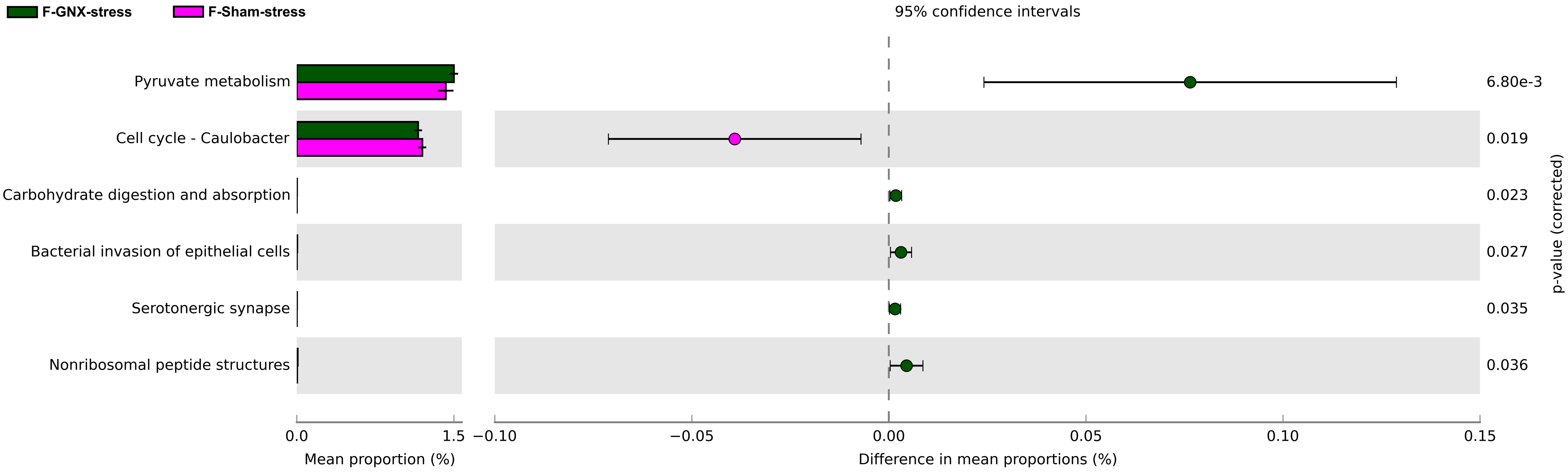

F-GNX-stress
F-Sham-stress
95% confidence intervals
Pyruvate metabolism
Cell cycle - Caulobacter
Carbohydrate digestion and absorption
Bacterial invasion of epithelial cells
Serotonergic synapse
Nonribosomal peptide structures
6.80e-3
0.019
0.023
0.027
0.035
0.036
p-value (corrected)
0.0
1.5
Mean proportion (%)
−0.10
−0.05
0.00
0.05
0.10
0.15
Difference in mean proportions (%)

Figure S16

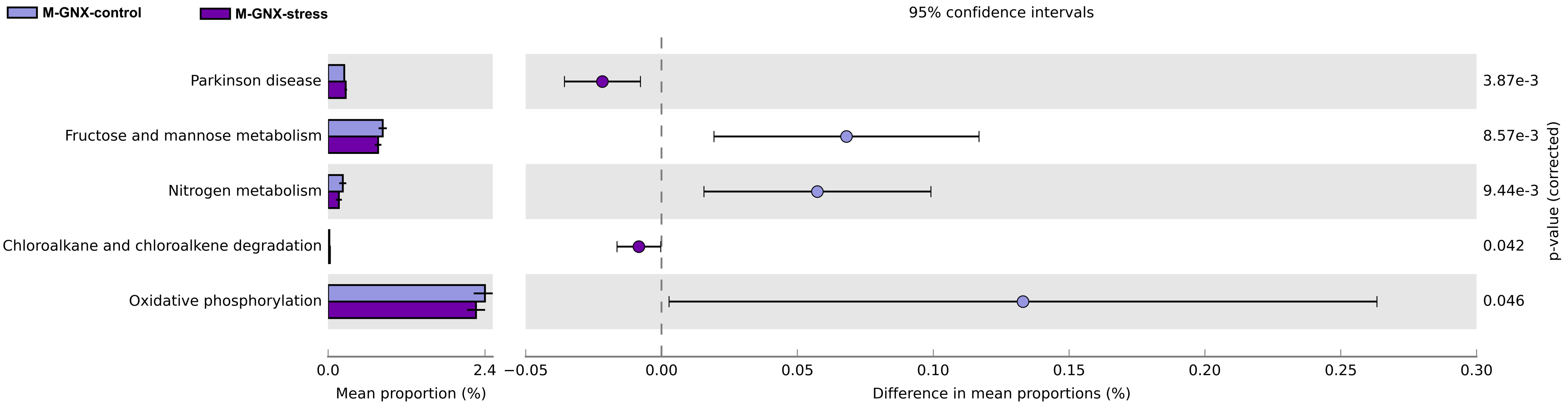
M-GNX-control
M-GNX-stress
95% confidence intervals
Parkinson disease
Fructose and mannose metabolism
Nitrogen metabolism
Chloroalkane and chloroalkene degradation
Oxidative phosphorylation
3.87e-3
8.57e-3
9.44e-3
0.042
0.046
p-value (corrected)
0.0
2.4
Mean proportion (%)
−0.05
0.00
0.05
0.10
0.15
0.20
0.25
0.30
Difference in mean proportions (%)

Figure S17

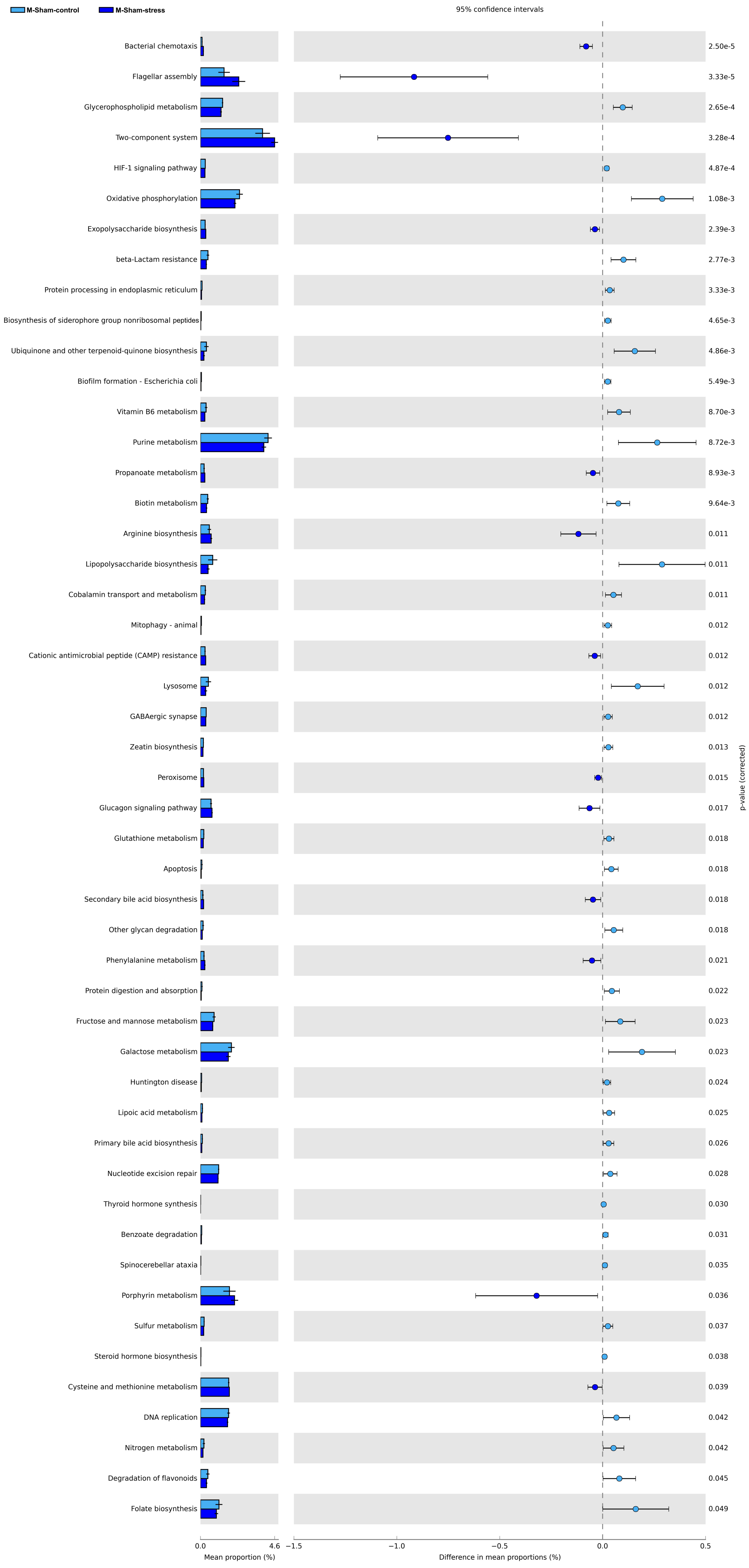
M-Sham-control
M-Sham-stress
95% confidence intervals
Bacterial chemotaxis
Flagellar assembly
Glycerophospholipid metabolism
Two-component system
HIF-1 signaling pathway
Oxidative phosphorylation
Exopolysaccharide biosynthesis
beta-Lactam resistance
Protein processing in endoplasmic reticulum
Biosynthesis of siderophore group nonribosomal peptides
Ubiquinone and other terpenoid-quinone biosynthesis
Biofilm formation - Escherichia coli
Vitamin B6 metabolism
Purine metabolism
Propanoate metabolism
Biotin metabolism
Arginine biosynthesis
Lipopolysaccharide biosynthesis
Cobalamin transport and metabolism
Mitophagy - animal
Cationic antimicrobial peptide (CAMP) resistance
Lysosome
GABAergic synapse
Zeatin biosynthesis
Peroxisome
Glucagon signaling pathway
Glutathione metabolism
Apoptosis
Secondary bile acid biosynthesis
Other glycan degradation
Phenylalanine metabolism
Protein digestion and absorption
Fructose and mannose metabolism
Galactose metabolism
Huntington disease
Lipoic acid metabolism
Primary bile acid biosynthesis
Nucleotide excision repair
Thyroid hormone synthesis
Benzoate degradation
Spinocerebellar ataxia
Porphyrin metabolism
Sulfur metabolism
Steroid hormone biosynthesis
Cysteine and methionine metabolism
DNA replication
Nitrogen metabolism
Degradation of flavonoids
Folate biosynthesis
2.50e-5
3.33e-5
2.65e-4
3.28e-4
4.87e-4
1.08e-3
2.39e-3
2.77e-3
3.33e-3
4.65e-3
4.86e-3
5.49e-3
8.70e-3
8.72e-3
8.93e-3
9.64e-3
0.011
0.011
0.011
0.012
0.012
0.012
0.012
0.013
0.015
0.017
0.018
0.018
0.018
0.018
0.021
0.022
0.023
0.023
0.024
0.025
0.026
0.028
0.030
0.031
0.035
0.036
0.037
0.038
0.039
0.042
0.042
0.045
0.049
p-value (corrected)
0.0
4.6
Mean proportion (%)
−1.5
−1.0
−0.5
0.0
0.5
Difference in mean proportions (%)

Figure S18

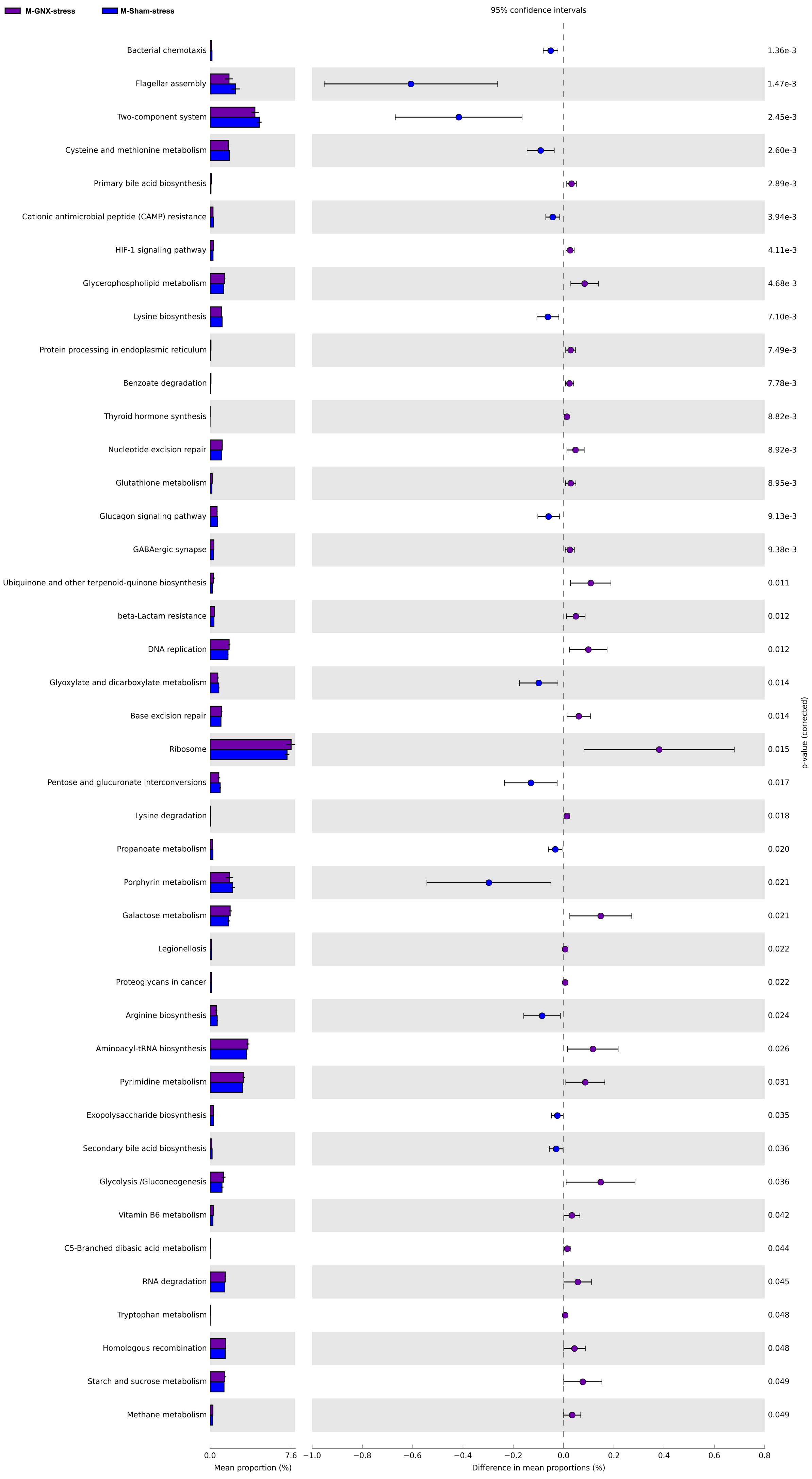

M-GNX-stress
M-Sham-stress
95% confidence intervals
Bacterial chemotaxis 1.36e-3
Flagellar assembly 1.47e-3
Two-component system 2.45e-3
Cysteine and methionine metabolism 2.60e-3
Primary bile acid biosynthesis 2.89e-3
Cationic antimicrobial peptide (CAMP) resistance 3.94e-3
HIF-1 signaling pathway 4.11e-3
Glycerophospholipid metabolism 4.68e-3
Lysine biosynthesis 7.10e-3
Protein processing in endoplasmic reticulum 7.49e-3
Benzoate degradation 7.78e-3
Thyroid hormone synthesis 8.82e-3
Nucleotide excision repair 8.92e-3
Glutathione metabolism 8.95e-3
Glucagon signaling pathway 9.13e-3
GABAergic synapse 9.38e-3
Ubiquinone and other terpenoid-quinone biosynthesis 0.011
beta-Lactam resistance 0.012
DNA replication 0.012
Glyoxylate and dicarboxylate metabolism 0.014
Base excision repair 0.014
Ribosome 0.015
Pentose and glucuronate interconversions 0.017
Lysine degradation 0.018
Propanoate metabolism 0.020
Porphyrin metabolism 0.021
Galactose metabolism 0.021
Legionellosis 0.022
Proteoglycans in cancer 0.022
Arginine biosynthesis 0.024
Aminoacyl-tRNA biosynthesis 0.026
Pyrimidine metabolism 0.031
Exopolysaccharide biosynthesis 0.035
Secondary bile acid biosynthesis 0.036
Glycolysis /Gluconeogenesis 0.036
Vitamin B6 metabolism 0.042
C5-Branched dibasic acid metabolism 0.044
RNA degradation 0.045
Tryptophan metabolism 0.048
Homologous recombination 0.048
Starch and sucrose metabolism 0.049
Methane metabolism 0.049
p-value (corrected)
0.0
7.6
Mean proportion (%)
−1.0 −0.8 −0.6 −0.4 −0.2 0.0 0.2 0.4 0.6 0.8
Difference in mean proportions (%)

Figure S19

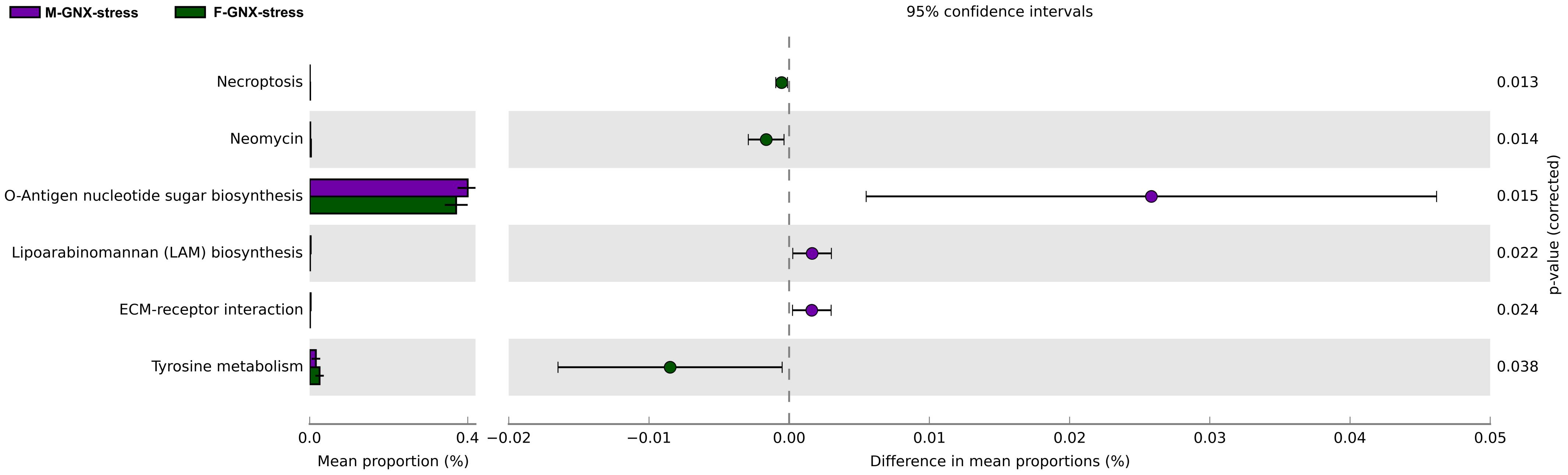
M-GNX-stress
F-GNX-stress
95% confidence intervals
Necroptosis
Neomycin
O-Antigen nucleotide sugar biosynthesis
Lipoarabinomannan (LAM) biosynthesis
ECM-receptor interaction
Tyrosine metabolism
0.013
0.014
0.015
0.022
0.024
0.038
p-value (corrected)
0.0
0.4
Mean proportion (%)
−0.02
−0.01
0.00
0.01
0.02
0.03
0.04
0.05
Difference in mean proportions (%)

Figure S20

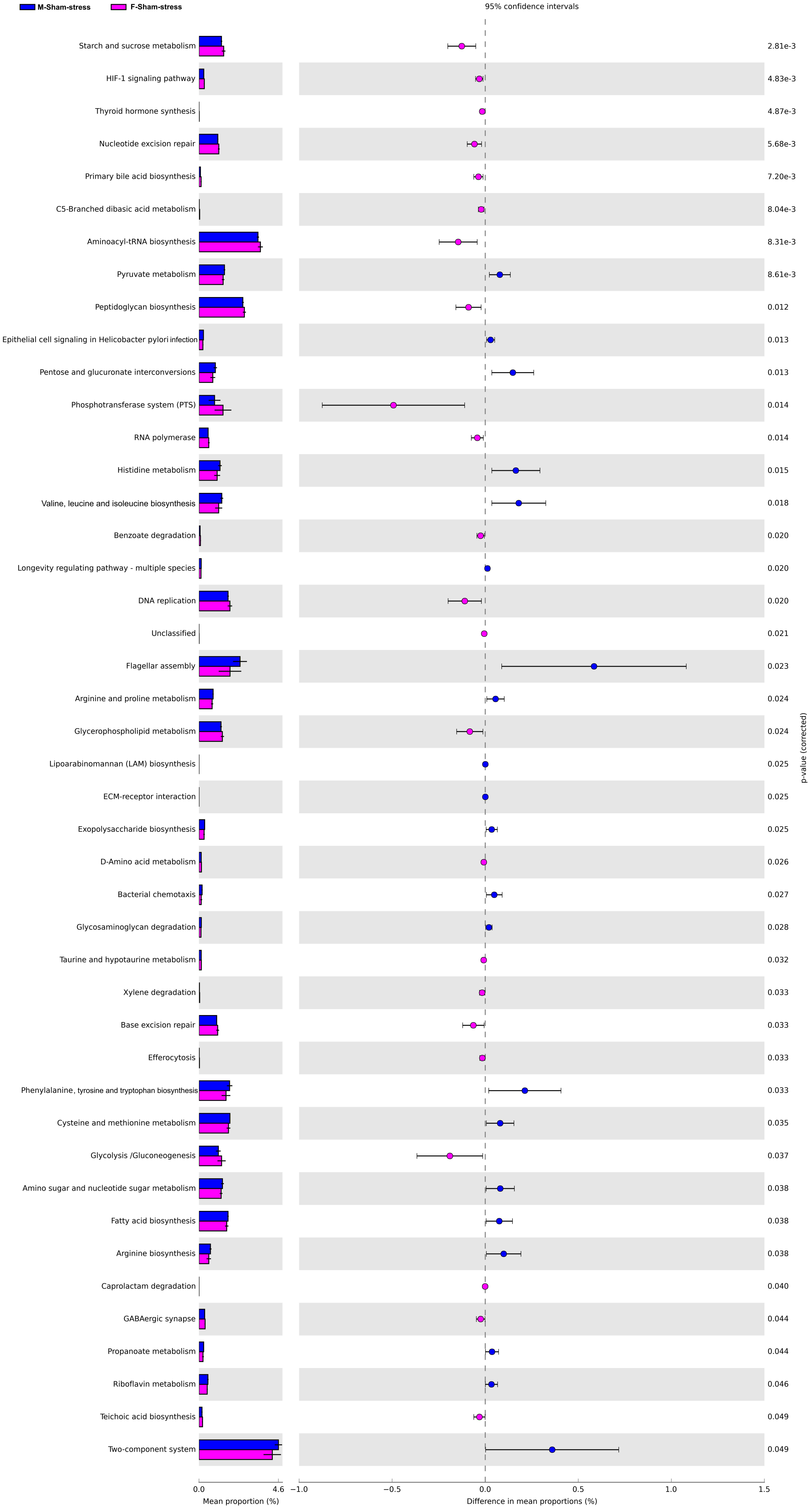
M-Sham-stress
F-Sham-stress
95% confidence intervals
Starch and sucrose metabolism 2.81e-3
HIF-1 signaling pathway 4.83e-3
Thyroid hormone synthesis 4.87e-3
Nucleotide excision repair 5.68e-3
Primary bile acid biosynthesis 7.20e-3
C5-Branched dibasic acid metabolism 8.04e-3
Aminoacyl-tRNA biosynthesis 8.31e-3
Pyruvate metabolism 8.61e-3
Peptidoglycan biosynthesis 0.012
Epithelial cell signaling in Helicobacter pylori infection 0.013
Pentose and glucuronate interconversions 0.013
Phosphotransferase system (PTS) 0.014
RNA polymerase 0.014
Histidine metabolism 0.015
Valine, leucine and isoleucine biosynthesis 0.018
Benzoate degradation 0.020
Longevity regulating pathway - multiple species 0.020
DNA replication 0.020
Unclassified 0.021
Flagellar assembly 0.023
Arginine and proline metabolism 0.024
Glycerophospholipid metabolism 0.024
Lipoarabinomannan (LAM) biosynthesis 0.025
ECM-receptor interaction 0.025
Exopolysaccharide biosynthesis 0.025
D-Amino acid metabolism 0.026
Bacterial chemotaxis 0.027
Glycosaminoglycan degradation 0.028
Taurine and hypotaurine metabolism 0.032
Xylene degradation 0.033
Base excision repair 0.033
Efferocytosis 0.033
Phenylalanine, tyrosine and tryptophan biosynthesis 0.033
Cysteine and methionine metabolism 0.035
Glycolysis /Gluconeogenesis 0.037
Amino sugar and nucleotide sugar metabolism 0.038
Fatty acid biosynthesis 0.038
Arginine biosynthesis 0.038
Caprolactam degradation 0.040
GABAergic synapse 0.044
Propanoate metabolism 0.044
Riboflavin metabolism 0.046
Teichoic acid biosynthesis 0.049
Two-component system 0.049
0.0
4.6
Mean proportion (%)
−1.0
−0.5
0.0
0.5
1.0
1.5
Difference in mean proportions (%)
p-value (corrected)

Figure S21

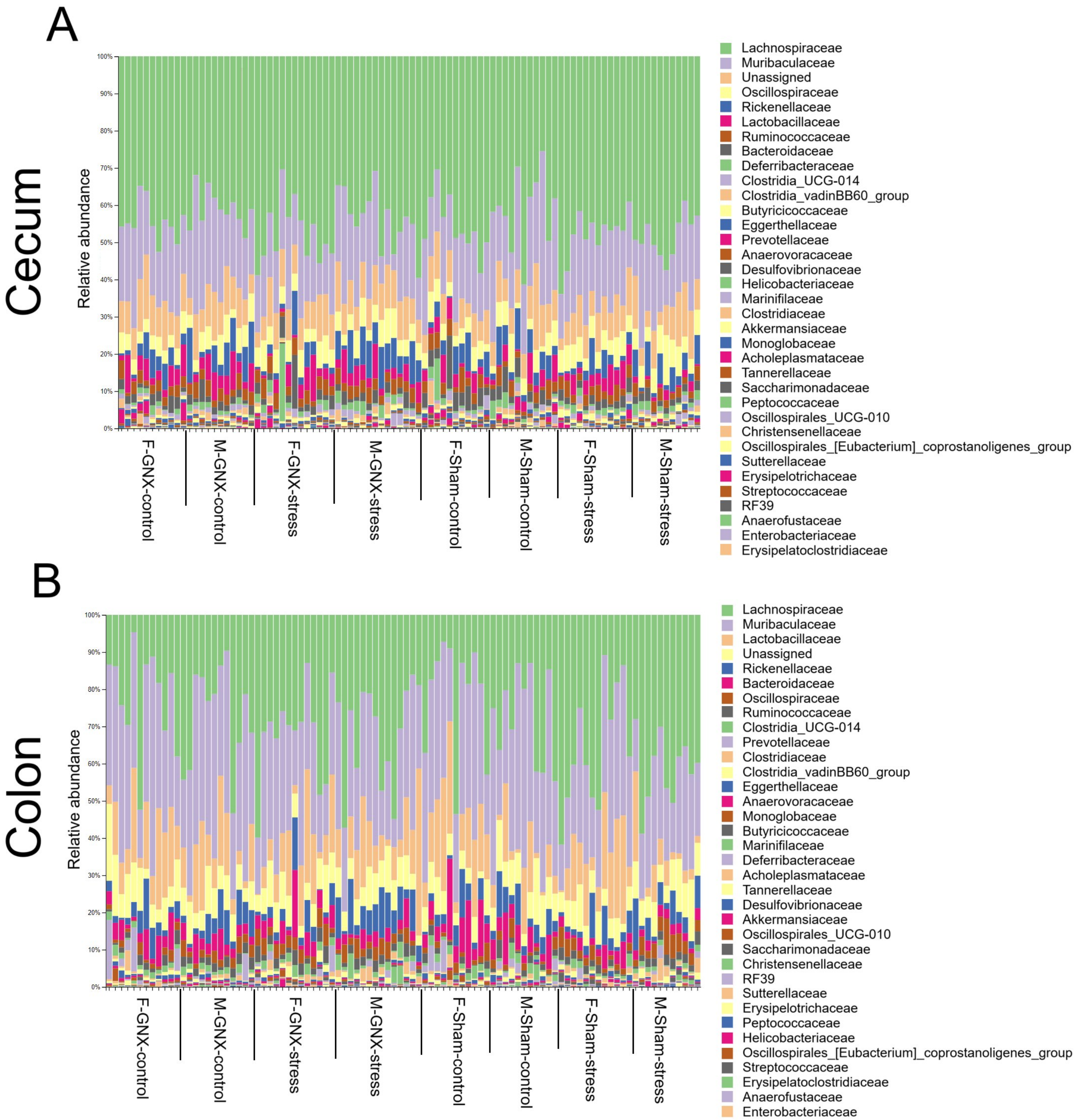
A
Cecum
Relative abundance
100%
90%
80%
70%
60%
50%
40%
30%
20%
10%
0%
F-GNX-control
M-GNX-control
F-GNX-stress
M-GNX-stress
F-Sham-control
M-Sham-control
F-Sham-stress
M-Sham-stress
Lachnospiraceae
Muribaculaceae
Unassigned
Oscillospiraceae
Rickenellaceae
Lactobacillaceae
Ruminococcaceae
Bacteroidaceae
Deferribacteraceae
Clostridia_UCG-014
Clostridia_vadinBB60_group
Butyricicoccaceae
Eggerthellaceae
Prevotellaceae
Anaerovoraceae
Desulfovibrionaceae
Helicobacteriaceae
Marinifilaceae
Clostridiaceae
Akkermansiaceae
Monoglobaceae
Acholeplasmataceae
Tannerellaceae
Saccharimonadaceae
Peptococcaceae
Oscillospirales_UCG-010
Christensenellaceae
Oscillospirales_[Eubacterium]_coprostanoligenes_group
Sutterellaceae
Erysipelotrichaceae
Streptococcaceae
RF39
Anaerofustaceae
Enterobacteriaceae
Erysipelatoclostridiaceae
B
Colon
Relative abundance
100%
90%
80%
70%
60%
50%
40%
30%
20%
10%
0%
F-GNX-control
M-GNX-control
F-GNX-stress
M-GNX-stress
F-Sham-control
M-Sham-control
F-Sham-stress
M-Sham-stress
Lachnospiraceae
Muribaculaceae
Lactobacillaceae
Unassigned
Rickenellaceae
Bacteroidaceae
Oscillospiraceae
Ruminococcaceae
Clostridia_UCG-014
Prevotellaceae
Clostridiaceae
Clostridia_vadinBB60_group
Eggerthellaceae
Anaerovoraceae
Monoglobaceae
Butyricicoccaceae
Marinifilaceae
Deferribacteraceae
Acholeplasmataceae
Tannerellaceae
Desulfovibrionaceae
Akkermansiaceae
Oscillospirales_UCG-010
Saccharimonadaceae
Christensenellaceae
RF39
Sutterellaceae
Erysipelotrichaceae
Peptococcaceae
Helicobacteriaceae
Oscillospirales_[Eubacterium]_coprostanoligenes_group
Streptococcaceae
Erysipelatoclostridiaceae
Anaerofustaceae
Enterobacteriaceae

Figure S22

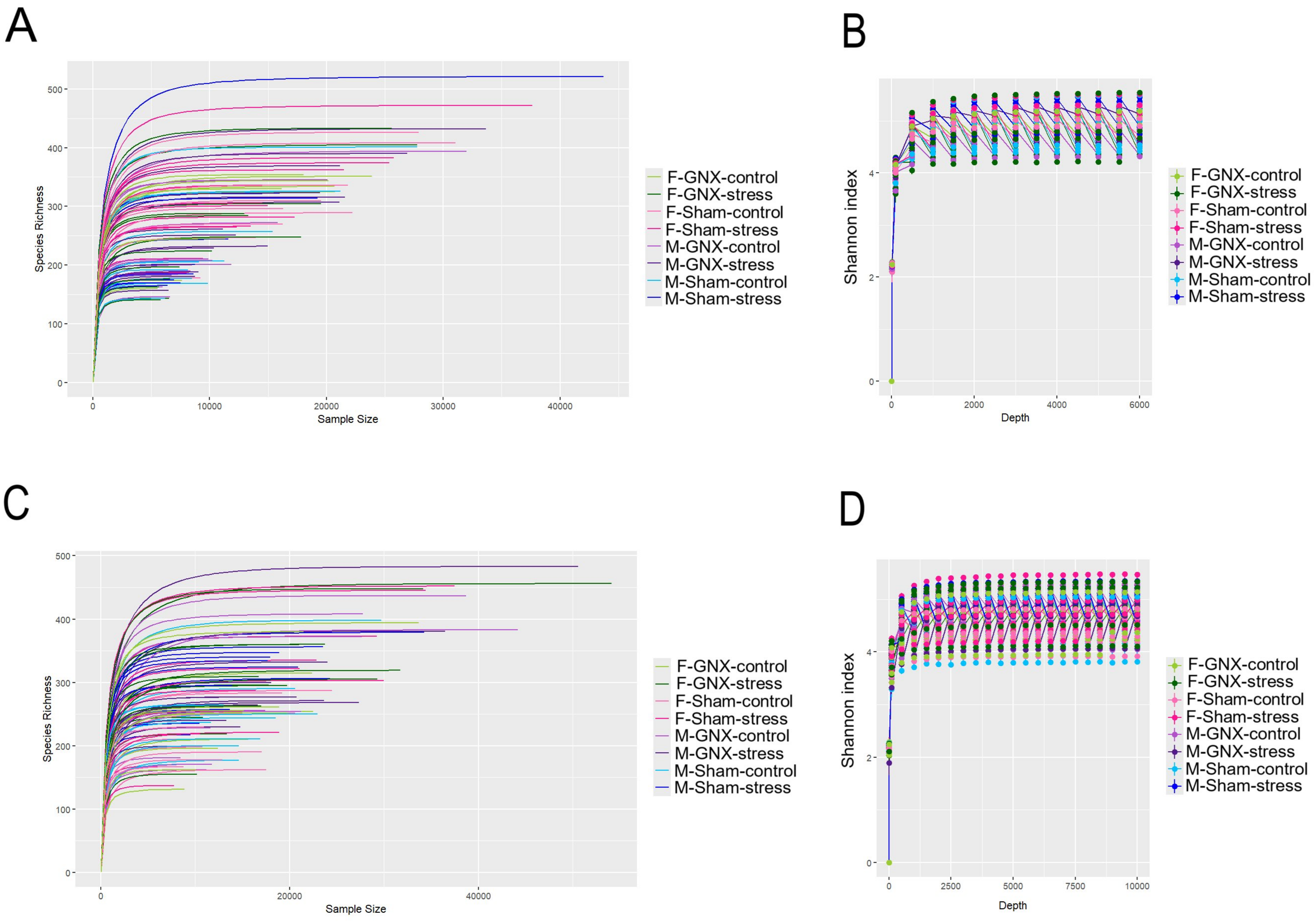
A
Species Richness
500
400
300
200
100
0
0
10000
20000
30000
40000
Sample Size
F-GNX-control
F-GNX-stress
F-Sham-control
F-Sham-stress
M-GNX-control
M-GNX-stress
M-Sham-control
M-Sham-stress
B
Shannon index
4
2
0
0
2000
4000
6000
Depth
F-GNX-control
F-GNX-stress
F-Sham-control
F-Sham-stress
M-GNX-control
M-GNX-stress
M-Sham-control
M-Sham-stress
C
Species Richness
500
400
300
200
100
0
0
20000
40000
Sample Size
F-GNX-control
F-GNX-stress
F-Sham-control
F-Sham-stress
M-GNX-control
M-GNX-stress
M-Sham-control
M-Sham-stress
D
Shannon index
4
2
0
0
2500
5000
7500
10000
Depth
F-GNX-control
F-GNX-stress
F-Sham-control
F-Sham-stress
M-GNX-control
M-GNX-stress
M-Sham-control
M-Sham-stress

Figure S23

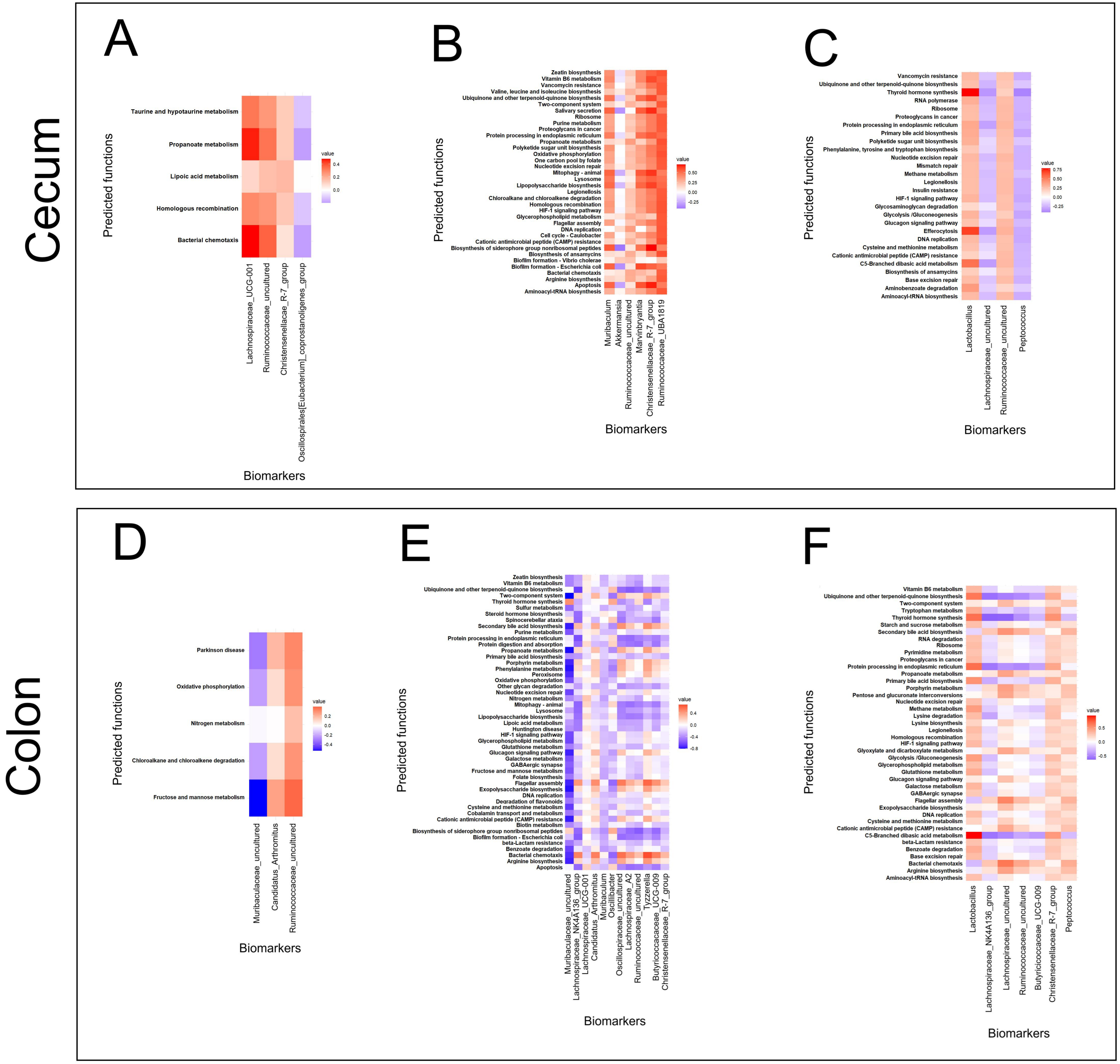

Cecum
A
Predicted functions
Taurine and hypotaurine metabolism
Propanoate metabolism
Lipoic acid metabolism
Homologous recombination
Bacterial chemotaxis
value
0.4
0.2
0.0
Lachnospiraceae_UCG-001
Ruminococcaceae_uncultured
Christensenellaceae_R-7_group
Oscillospirales[Eubacterium]_coprostanoligenes_group
Biomarkers
B
Predicted functions
Zeatin biosynthesis
Vitamin B6 metabolism
Vancomycin resistance
Valine, leucine and isoleucine biosynthesis
Ubiquinone and other terpenoid-quinone biosynthesis
Two-component system
Salivary secretion
Ribosome
Purine metabolism
Proteoglycans in cancer
Protein processing in endoplasmic reticulum
Propanoate metabolism
Polyketide sugar unit biosynthesis
Oxidative phosphorylation
One carbon pool by folate
Nucleotide excision repair
Mitophagy - animal
Lysosome
Lipopolysaccharide biosynthesis
Legionellosis
Chloroalkane and chloroalkene degradation
Homologous recombination
HIF-1 signaling pathway
Glycerophospholipid metabolism
Flagellar assembly
DNA replication
Cell cycle - Caulobacter
Cationic antimicrobial peptide (CAMP) resistance
Biosynthesis of siderophore group nonribosomal peptides
Biosynthesis of ansamycins
Biofilm formation - Vibrio cholerae
Biofilm formation - Escherichia coli
Bacterial chemotaxis
Arginine biosynthesis
Apoptosis
Aminoacyl-tRNA biosynthesis
value
0.50
0.25
0.00
-0.25
Muribaculum
Akkermansia
Ruminococcaceae_uncultured
Marvinbryantia
Christensenellaceae_R-7_group
Ruminococcaceae_UBA1819
Biomarkers
C
Predicted functions
Vancomycin resistance
Ubiquinone and other terpenoid-quinone biosynthesis
Thyroid hormone synthesis
RNA polymerase
Ribosome
Proteoglycans in cancer
Protein processing in endoplasmic reticulum
Primary bile acid biosynthesis
Polyketide sugar unit biosynthesis
Phenylalanine, tyrosine and tryptophan biosynthesis
Nucleotide excision repair
Mismatch repair
Methane metabolism
Legionellosis
Insulin resistance
HIF-1 signaling pathway
Glycosaminoglycan degradation
Glycolysis /Gluconeogenesis
Glucagon signaling pathway
Efferocytosis
DNA replication
Cysteine and methionine metabolism
Cationic antimicrobial peptide (CAMP) resistance
C5-Branched dibasic acid metabolism
Biosynthesis of ansamycins
Base excision repair
Aminobenzoate degradation
Aminoacyl-tRNA biosynthesis
value
0.75
0.50
0.25
0.00
-0.25
Lactobacillus
Lachnospiraceae_uncultured
Ruminococcaceae_uncultured
Peptococcus
Biomarkers
Colon
D
Predicted functions
Parkinson disease
Oxidative phosphorylation
Nitrogen metabolism
Chloroalkane and chloroalkene degradation
Fructose and mannose metabolism
value
0.2
0.0
-0.2
-0.4
Muribaculaceae_uncultured
Candidatus_Arthromitus
Ruminococcaceae_uncultured
Biomarkers
E
Predicted functions
Zeatin biosynthesis
Vitamin B6 metabolism
Ubiquinone and other terpenoid-quinone biosynthesis
Two-component system
Thyroid hormone synthesis
Sulfur metabolism
Steroid hormone biosynthesis
Spinocerebellar ataxia
Secondary bile acid biosynthesis
Purine metabolism
Protein processing in endoplasmic reticulum
Protein digestion and absorption
Propanoate metabolism
Primary bile acid biosynthesis
Porphyrin metabolism
Phenylalanine metabolism
Peroxisome
Oxidative phosphorylation
Other glycan degradation
Nucleotide excision repair
Nitrogen metabolism
Mitophagy - animal
Lysosome
Lipopolysaccharide biosynthesis
Lipoic acid metabolism
Huntington disease
HIF-1 signaling pathway
Glycerophospholipid metabolism
Glutathione metabolism
Glucagon signaling pathway
Galactose metabolism
GABAergic synapse
Fructose and mannose metabolism
Folate biosynthesis
Flagellar assembly
Exopolysaccharide biosynthesis
DNA replication
Degradation of flavonoids
Cysteine and methionine metabolism
Cobalamin transport and metabolism
Cationic antimicrobial peptide (CAMP) resistance
Biotin metabolism
Biosynthesis of siderophore group nonribosomal peptides
Biofilm formation - Escherichia coli
beta-Lactam resistance
Benzoate degradation
Bacterial chemotaxis
Arginine biosynthesis
Apoptosis
value
0.4
0.0
-0.4
-0.8
Muribaculaceae_uncultured
Lachnospiraceae_NK4A136_group
Lachnospiraceae_UCG-001
Candidatus_Arthromitus
Muribaculum
Oscillibacter
Oscillospiraceae_uncultured
Lachnospiraceae_A2
Ruminococcaceae_uncultured
Tyzzerella
Butyricicoccaceae_UCG-009
Christensenellaceae_R-7_group
Biomarkers
F
Predicted functions
Vitamin B6 metabolism
Ubiquinone and other terpenoid-quinone biosynthesis
Two-component system
Tryptophan metabolism
Thyroid hormone synthesis
Starch and sucrose metabolism
Secondary bile acid biosynthesis
RNA degradation
Ribosome
Pyrimidine metabolism
Proteoglycans in cancer
Protein processing in endoplasmic reticulum
Propanoate metabolism
Primary bile acid biosynthesis
Porphyrin metabolism
Pentose and glucuronate interconversions
Nucleotide excision repair
Methane metabolism
Lysine degradation
Lysine biosynthesis
Legionellosis
Homologous recombination
HIF-1 signaling pathway
Glyoxylate and dicarboxylate metabolism
Glycolysis /Gluconeogenesis
Glycerophospholipid metabolism
Glutathione metabolism
Glucagon signaling pathway
Galactose metabolism
GABAergic synapse
Flagellar assembly
Exopolysaccharide biosynthesis
DNA replication
Cysteine and methionine metabolism
Cationic antimicrobial peptide (CAMP) resistance
C5-Branched dibasic acid metabolism
beta-Lactam resistance
Benzoate degradation
Base excision repair
Bacterial chemotaxis
Arginine biosynthesis
Aminoacyl-tRNA biosynthesis
value
0.5
0.0
-0.5
Lactobacillus
Lachnospiraceae_NK4A136_group
Lachnospiraceae_uncultured
Ruminococcaceae_uncultured
Butyricicoccaceae_UCG-009
Christensenellaceae_R-7_group
Peptococcus
Biomarkers

Figure S24

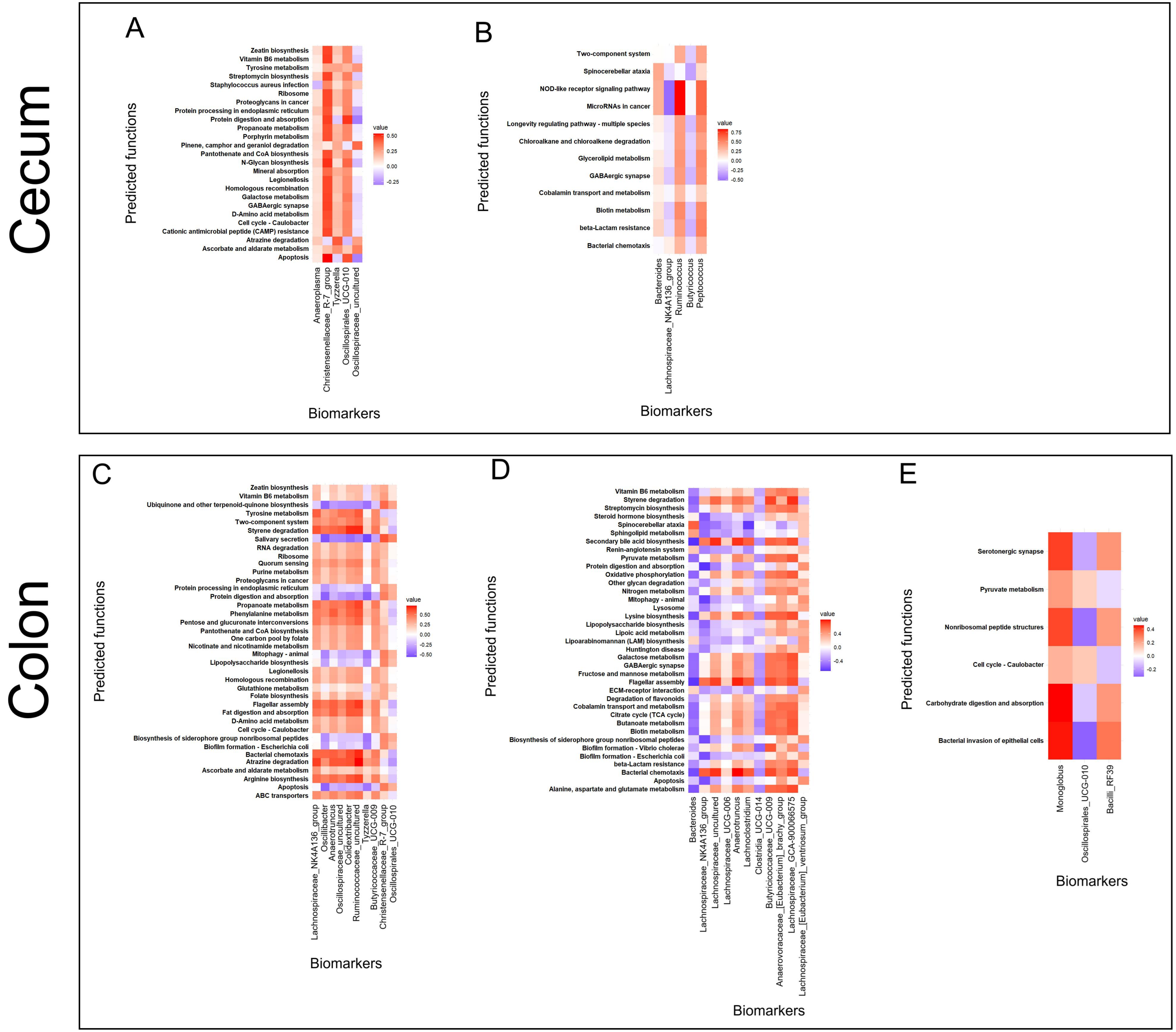

Cecum
A
Predicted functions
Zeatin biosynthesis
Vitamin B6 metabolism
Tyrosine metabolism
Streptomycin biosynthesis
Staphylococcus aureus infection
Ribosome
Proteoglycans in cancer
Protein processing in endoplasmic reticulum
Protein digestion and absorption
Propanoate metabolism
Porphyrin metabolism
Pinene, camphor and geraniol degradation
Pantothenate and CoA biosynthesis
N-Glycan biosynthesis
Mineral absorption
Legionellosis
Homologous recombination
Galactose metabolism
GABAergic synapse
D-Amino acid metabolism
Cell cycle - Caulobacter
Cationic antimicrobial peptide (CAMP) resistance
Atrazine degradation
Ascorbate and aldarate metabolism
Apoptosis
Anaeroplasma
Christensenellaceae_R-7_group
Tyzzerella
Oscillospirales_UCG-010
Oscillospiraceae_uncultured
Biomarkers
value
0.50
0.25
0.00
-0.25
B
Predicted functions
Two-component system
Spinocerebellar ataxia
NOD-like receptor signaling pathway
MicroRNAs in cancer
Longevity regulating pathway - multiple species
Chloroalkane and chloroalkene degradation
Glycerolipid metabolism
GABAergic synapse
Cobalamin transport and metabolism
Biotin metabolism
beta-Lactam resistance
Bacterial chemotaxis
Bacteroides
Lachnospiraceae_NK4A136_group
Ruminococcus
Butyricicoccus
Peptococcus
Biomarkers
value
0.75
0.50
0.25
0.00
-0.25
-0.50
Colon
C
Predicted functions
Zeatin biosynthesis
Vitamin B6 metabolism
Ubiquinone and other terpenoid-quinone biosynthesis
Tyrosine metabolism
Two-component system
Styrene degradation
Salivary secretion
RNA degradation
Ribosome
Quorum sensing
Purine metabolism
Proteoglycans in cancer
Protein processing in endoplasmic reticulum
Protein digestion and absorption
Propanoate metabolism
Phenylalanine metabolism
Pentose and glucuronate interconversions
Pantothenate and CoA biosynthesis
One carbon pool by folate
Nicotinate and nicotinamide metabolism
Mitophagy - animal
Lipopolysaccharide biosynthesis
Legionellosis
Homologous recombination
Glutathione metabolism
Folate biosynthesis
Flagellar assembly
Fat digestion and absorption
D-Amino acid metabolism
Cell cycle - Caulobacter
Biosynthesis of siderophore group nonribosomal peptides
Biofilm formation - Escherichia coli
Bacterial chemotaxis
Atrazine degradation
Ascorbate and aldarate metabolism
Arginine biosynthesis
Apoptosis
ABC transporters
Lachnospiraceae_NK4A136_group
Oscillibacter
Anaerotruncus
Oscillospiraceae_uncultured
Colidextribacter
Ruminococcaceae_uncultured
Tyzzerella
Butyricicoccaceae_UCG-009
Christensenellaceae_R-7_group
Oscillospirales_UCG-010
Biomarkers
value
0.50
0.25
0.00
-0.25
-0.50
D
Predicted functions
Vitamin B6 metabolism
Styrene degradation
Streptomycin biosynthesis
Steroid hormone biosynthesis
Spinocerebellar ataxia
Sphingolipid metabolism
Secondary bile acid biosynthesis
Renin-angiotensin system
Pyruvate metabolism
Protein digestion and absorption
Oxidative phosphorylation
Other glycan degradation
Nitrogen metabolism
Mitophagy - animal
Lysosome
Lysine biosynthesis
Lipopolysaccharide biosynthesis
Lipoic acid metabolism
Lipoarabinomannan (LAM) biosynthesis
Huntington disease
Galactose metabolism
GABAergic synapse
Fructose and mannose metabolism
Flagellar assembly
ECM-receptor interaction
Degradation of flavonoids
Cobalamin transport and metabolism
Citrate cycle (TCA cycle)
Butanoate metabolism
Biotin metabolism
Biosynthesis of siderophore group nonribosomal peptides
Biofilm formation - Vibrio cholerae
Biofilm formation - Escherichia coli
beta-Lactam resistance
Bacterial chemotaxis
Apoptosis
Alanine, aspartate and glutamate metabolism
Bacteroides
Lachnospiraceae_NK4A136_group
Lachnospiraceae_uncultured
Lachnospiraceae_UCG-006
Anaerotruncus
Lachnoclostridium
Clostridia_UCG-014
Butyricicoccaceae_UCG-009
Anaerovoracaceae_[Eubacterium]_brachy_group
Lachnospiraceae_GCA-900066575
Lachnospiraceae_[Eubacterium]_ventriosum_group
Biomarkers
value
0.4
0.0
-0.4
E
Predicted functions
Serotonergic synapse
Pyruvate metabolism
Nonribosomal peptide structures
Cell cycle - Caulobacter
Carbohydrate digestion and absorption
Bacterial invasion of epithelial cells
Monoglobus
Oscillospirales_UCG-010
Bacilli_RF39
Biomarkers
value
0.4
0.2
0.0
-0.2
