# Supplementary material for: Impact of Sex, Gonadectomy, and Repeated Restraint Stress on Gut Microbiome in Mice
Source: Mol Neurobiol. 2025 Nov 19;63(1):80. doi: 10.1007/s12035-025-05305-6 (PMC12627175; doi:10.1007/s12035-025-05305-6)
Supplement: Supplementary file 7 — (PDF 651 KB) [file 12035_2025_5305_MOESM7_ESM.pdf]

Molecular Neurobiology

Impact of sex, gonadectomy and repeated restraint stress on gut microbiome in mice

Chahrazed Mekadim[1]*, Jakub Mrázek[1], Martin Vodička[2], Peter Ergang[2], Kateřina Olša Fliegerová[1], Tiziana Maria Mahayri[1], Kallayanee Chawengsaksophak[4], Jiří Pácha[2,3]

1 Laboratory of Anaerobic Microbiology, Institute of Animal Physiology and Genetics, Czech Academy of Sciences, v.v.i., Prague, Czech Republic.
2 Laboratory of Epithelial Physiology, Institute of Physiology, Czech Academy of Sciences, v.v.i., Prague, Czech Republic.
3 Department of Physiology, Faculty of Science, Charles University, Prague, Czech Republic.
4Laboratory of Cell Differentiation, Institute of Molecular Genetics, Czech Academy of Sciences, v.v.i., Prague, Czech Republic.

* Correspondence:
Chahrazed Mekadim
mekadim@iapg.cas.cz

Table S6 Summary table showing number of identified biomarkers and predicted functions in cecal and colonic microbiome for each relevant group. Common biomarkers and predicted functions between cecal and colonic microbiome were indicated for each group.

		F-Sham		F-GNX		F-stress		GNX-stress		Sham-stress	
		stress	control	stress	control	Sham	GNX	M	F	M	F
Number of identified biomarker	cecum	2	3	2	3	1	1	1	2	5	0
	colon	8	3	8	2	1	1	1	4	5	3
Number of upregulated functions	cecum	4	8	9	16	/	/	30	8	11	12
	colon	6	31	14	24	1	5	3	3	21	23
common biomarker in cecum and colon		Lachnospiraceae_NK4A136_group	Bacteroides	Oscillospiraceae_uncultured	Christensenellaceae_R-7_group, Oscillospirales_UCG-010	/	Monoglobus	Alistipes	Anaeroplasma, Monoglobus	/	Lactobacillus
common functions in cecum and colon		Bacterial chemotaxis	beta-Lactam resistance	Propanoate metabolism	Apoptosis	/	/	O-Antigen nucleotide sugar biosynthesis	/	Arginine biosynthesis	Efferocytosis
			Biotin metabolism	Ascorbate and aldarate metabolism	Cell cycle - Caulobacter					Histidine metabolism	Glycerophospholipid metabolism
			GABAergic synapse	Atrazine degradation	D-Amino acid metabolism					Riboflavin metabolism	Glycolysis /Gluconeogenesis
			Spinocerebellar ataxia	Tyrosine metabolism	Homologous recombination					Epithelial cell signaling in Helicobacter pylori infection	HIF-1 signaling pathway
					Legionellosis					Phenylalanine, tyrosine and tryptophan biosynthesis	Histidine metabolism
					Pantothenate and CoA biosynthesis					Pyruvate metabolism	Phosphotransferase system (PTS)
					Protein digestion and absorption					Valine, leucine and isoleucine biosynthesis	Primary bile acid biosynthesis
					Protein processing in endoplasmic reticulum					Fatty acid biosynthesis	Pyruvate metabolism
					Proteoglycans in cancer						Riboflavin metabolism
					Ribosome						Thyroid hormone synthesis
					Vitamin B6 metabolism						Xylene degradation
					Zeatin biosynthesis						

		M-Sham		M-GNX		M-stress	
		stress	control	stress	control	Sham	GNX
Number of identified biomarker	cecum	1	5	2	2	2	2
	colon	9	3	2	1	5	2
Number of upregulated functions	cecum	10	26	2	3	6	22
	colon	13	36	2	3	14	28
common biomarker in cecum and colon		/	Muribaculum, Christensenellaceae_R-7_group	Ruminococcaceae_uncultured	/	Lachnospiraceae_uncultured, Peptococcus	Lactobacillus
common functionsin cecum and colon		Bacterial chemotaxis<br>Flagellar assembly<br>Two-component system<br>Arginine biosynthesis<br>Propanoate metabolism	Apoptosis<br>Bacterial chemotaxis<br>Biofilm formation - Escherichia coli<br>Biosynthesis of siderophore group nonribosomal peptides<br>Cationic antimicrobial peptide (CAMP) resistance<br>DNA replication<br>Flagellar assembly<br>Glycerophospholipid metabolism<br>HIF-1 signaling pathway<br>Lysosome<br>Mitophagy - animal<br>Nucleotide excision repair<br>Oxidative phosphorylation<br>Propanoate metabolism<br>Protein processing in endoplasmic reticulum<br>Purine metabolism<br>Ubiquinone and other terpenoid-quinone biosynthesis<br>Vitamin B6 metabolism<br>Zeatin biosynthesis	/	/	Cationic antimicrobial peptide (CAMP) resistance<br>Glucagon signaling pathway	Aminoacyl-tRNA biosynthesis<br>Base excision repair<br>C5-Branched dibasic acid metabolism<br>Cysteine and methionine metabolism<br>DNA replication<br>Glycolysis /Gluconeogenesis<br>Glycosaminoglycan degradation<br>HIF-1 signaling pathway<br>Legionellosis<br>Methane metabolism<br>Nucleotide excision repair<br>Primary bile acid biosynthesis<br>Protein processing in endoplasmic reticulum<br>Proteoglycans in cancer<br>Ribosome<br>RNA polymerase<br>Thyroid hormone synthesis<br>Ubiquinone and other terpenoid-quinone biosynthesis
